# Development of a standardized and validated flow cytometry approach for monitoring of innate myeloid immune cells in human blood

**DOI:** 10.3389/fimmu.2022.935879

**Published:** 2022-09-14

**Authors:** Kyra van der Pan, Sandra de Bruin-Versteeg, Daniela Damasceno, Alejandro Hernández-Delgado, Alita J. van der Sluijs-Gelling, Wouter B. L. van den Bossche, Inge F. de Laat, Paula Díez, Brigitta A. E. Naber, Annieck M. Diks, Magdalena A. Berkowska, Bas de Mooij, Rick J. Groenland, Fenna J. de Bie, Indu Khatri, Sara Kassem, Anniek L. de Jager, Alesha Louis, Julia Almeida, Jacqueline A. M. van Gaans-van den Brink, Alex-Mikael Barkoff, Qiushui He, Gerben Ferwerda, Pauline Versteegen, Guy A. M. Berbers, Alberto Orfao, Jacques J. M. van Dongen, Cristina Teodosio

**Affiliations:** ^1^ Department of Immunology, Leiden University Medical Center, Leiden, Netherlands; ^2^ Translational and Clinical Research Program, Cancer Research Center (IBMCC; University of Salamanca - CSIC), Cytometry Service, NUCLEUS, Department of Medicine, University of Salamanca (Universidad de Salamanca, and Institute of Biomedical Research of Salamanca (IBSAL), Salamanca, Spain; ^3^ Department of Immunology, Department of Neurosurgery, Brain Tumor Center, Erasmus Medical Center, University Medical Center Rotterdam, Rotterdam, Netherlands; ^4^ Centre for Infectious Disease Control, National Institute for Public Health and the Environment (RIVM), Bilthoven, Netherlands; ^5^ Institute of Biomedicine, Research Center for Infections and Immunity, University of Turku (UTU), Turku, Finland; ^6^ Section of Paediatric Infectious Diseases, Laboratory of Medical Immunology, Radboud Institute for Molecular Life Sciences, Nijmegen, Netherlands

**Keywords:** immune-monitoring, flow cytometry, innate myeloid cells, age-related reference values, standardization

## Abstract

Innate myeloid cell (IMC) populations form an essential part of innate immunity. Flow cytometric (FCM) monitoring of IMCs in peripheral blood (PB) has great clinical potential for disease monitoring due to their role in maintenance of tissue homeostasis and ability to sense micro-environmental changes, such as inflammatory processes and tissue damage. However, the lack of standardized and validated approaches has hampered broad clinical implementation. For accurate identification and separation of IMC populations, 62 antibodies against 44 different proteins were evaluated. In multiple rounds of EuroFlow-based design-testing-evaluation-redesign, finally 16 antibodies were selected for their non-redundancy and separation power. Accordingly, two antibody combinations were designed for fast, sensitive, and reproducible FCM monitoring of IMC populations in PB in clinical settings (11-color; 13 antibodies) and translational research (14-color; 16 antibodies). Performance of pre-analytical and analytical variables among different instruments, together with optimized post-analytical data analysis and reference values were assessed. Overall, 265 blood samples were used for design and validation of the antibody combinations and *in vitro* functional assays, as well as for assessing the impact of sample preparation procedures and conditions. The two (11- and 14-color) antibody combinations allowed for robust and sensitive detection of 19 and 23 IMC populations, respectively. Highly reproducible identification and enumeration of IMC populations was achieved, independently of anticoagulant, type of FCM instrument and center, particularly when database/software-guided automated (*vs.* manual “expert-based”) gating was used. Whereas no significant changes were observed in identification of IMC populations for up to 24h delayed sample processing, a significant impact was observed in their absolute counts after >12h delay. Therefore, accurate identification and quantitation of IMC populations requires sample processing on the same day. Significantly different counts were observed in PB for multiple IMC populations according to age and sex. Consequently, PB samples from 116 healthy donors (8-69 years) were used for collecting age and sex related reference values for all IMC populations. In summary, the two antibody combinations and FCM approach allow for rapid, standardized, automated and reproducible identification of 19 and 23 IMC populations in PB, suited for monitoring of innate immune responses in clinical and translational research settings.

## Graphical Abstract

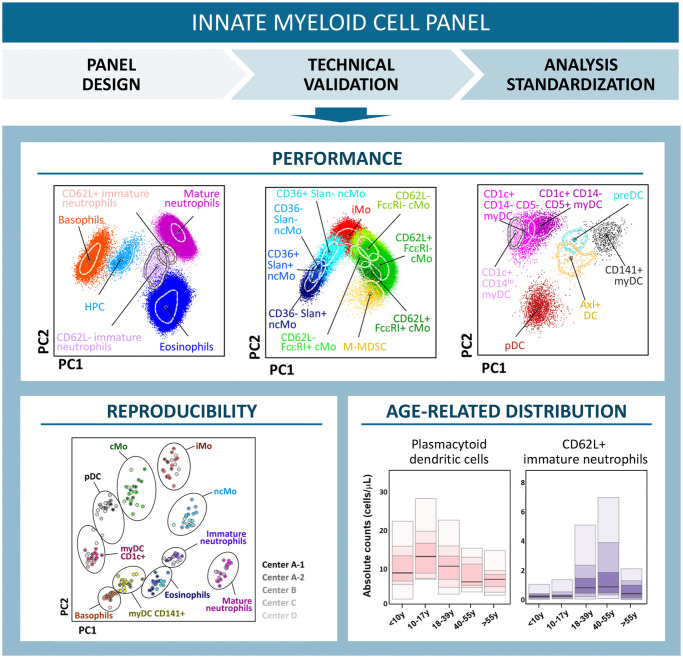

## Introduction

Monocytes, dendritic cells (DCs) and granulocytes, together also called innate myeloid cells (IMCs), play key roles in multiple different processes related to maintenance of tissue homeostasis, including sensing of tissue damage, induction and/or resolution of inflammation, antigen presentation and pathogen eradication (1–9). While some of these cell populations, such as mast cells and macrophages, are merely tissue-resident, others like monocytes, DCs, basophils, eosinophils and neutrophils circulate *via* peripheral blood (PB) with the ability to sense micro-environmental changes (such as inflammatory processes) and migrate to tissues where they modulate local responses in both physiological and disease conditions (10–12). This great plasticity and functional heterogeneity of IMCs renders them into ideal candidates for monitoring disturbances in body homeostasis at the systemic level, e.g. in PB. Consequently, the clinical utility of monitoring IMCs in PB for diagnosis, staging, prognostic assessment and/or evaluating response to treatment in multiple disease conditions has been demonstrated previously (9, 13–24).

However, monitoring IMCs for translational research and diagnostic patient care is currently hampered by the lack of standardized approaches. This includes the absence of immunophenotypic consensus criteria for the definition of the distinct IMC subsets, due to their great heterogeneity and plasticity (25–31) and the limited availability of lineage-specific proteins, which have led to the introduction of e.g., marker cocktails for lineage exclusion and highly variable strategies and/or extensive sets of markers for correct identification of the target populations (25–27, 29, 31–34). Additionally, new monocytes and DCs have been identified, leading to progressively more complex antibody panels and data analysis procedures. For example, new subsets of classical (cMo) and non-classical (ncMo) monocytes have recently been defined based on the expression pattern of CD9, CD62L, CD93 and/or FcϵRI and CD9, CD36 and Slan, respectively (35–39). Likewise, CD1c^+^ myeloid dendritic cells (myDCs) are now known to include different functional subsets, that can be identified based on CD14 expression (CD14^-^ non-inflammatory and a CD14^lo^ pro-inflammatory CD1c^+^ myDC population) (40) and CD5: CD5^hi^ CD1c^+^ myDCs with higher ability to migrate to the lymph nodes and induce T cell proliferation, and CD5^-^ CD1c^+^ myDCs with a closer functional profile to monocytes (41, 42). In addition, the new subset of Axl^+^ and SIGLEC6^+^ DC (Axl^+^ DCs) has been described recently, which was previously included in the plasmacytoid dendritic cell (pDC) population and exhibits mixed gene expression and functional profiles between pDCs and myDCs (40). In parallel, a new population of DC precursors has been described, co-expressing CD34^int^ and CD100^hi^, with the ability to generate *in vitro* both CD1c^+^ and CD141^+^ myDCs (31, 40, 43).

In recent years, different 8-12 to 38 color panels have been designed and proposed for monitoring monocytic and DC populations in PB by flow cytometry (FCM) and mass cytometry, respectively (26, 44–46). However, careful analysis of these FCM antibody panels shows that they typically include multiple redundant markers for defining IMC populations (e.g., CD123, CD303 and/or CD304 for identification of pDCs) and/or they require the use of antibody cocktails for exclusion (e.g., “dump channel”) of e.g. lymphoid cells, which prevent the addition of other relevant markers (29, 45). In contrast, a previously described 38-color mass cytometry antibody panel allows identification and characterization of virtually all monocyte, monocyte-derived macrophage, DC and myeloid-derived suppressor cell populations (47). However, mass cytometry is not readily available in many centers and, most importantly, has a very low throughput (250-350 cells/sec) and limited levels standardization, which limit its use in clinical settings. Furthermore, none of the previously reported antibody panels allow identification of the recently described DC and monocyte populations. At the same time these antibody panels did not use standardized and validated procedures for antibody panel design and data analysis in a multicentric setting, and failed to provide age-matched related ranges for the IMC populations (26, 44–46, 48, 49).

Here we designed and validated two (11- and 14-color) antibody panels for standardized, automated, and reproducible identification of 19 to 23 IMC populations in human blood by FCM, and provide age and sex-matched reference values for more objective interpretation of altered IMC profiles in multicentric clinical settings. Ultimately, the antibody panels developed will allow to set a new benchmark for IMC in both clinical and translational research settings.

## Material and methods

### Samples

For this study, 261 PB samples (195 ethylenediaminetetraacetic acid -EDTA- and 66 sodium heparin-anticoagulated) obtained from 205 healthy donors (HD) were evaluated. From them, 242 samples from 197 donors were used for antibody panel development and evaluation (72 men, 118 women and 7 donors lacking sex information, with median age of 32 years -y- ranging from 5y to 99y). For assessment of the technical performance of the antibody panels, construction & validation of the reference database for automated gating (50) 57 samples from 48 donors were used (20 men, 25 women; median age 38y; range: 5y – 85y; of note, sex information was not available for 3 donors). A total of 116 samples from 67 women and 45 men (unknown sex in 4 donors) with median age 30y (range: 8y-69y) were processed for defining age- and sex-related normal reference ranges. Additionally, 4 cord blood (CB) samples collected in EDTA were also included for the study of infrequent populations in steady-state PB, which are reported to be increased in CB (e.g., myeloid-derived suppressor cells -MDSC-, immature neutrophils). All samples were collected after informed consent was provided by each donor according to the Declaration of Helsinki and the guidelines of the local ethics committees and review boards. Of note, this study includes pre-vaccination samples collected and processed in the context of the Dutch ‘BERT study’, which was initiated by the Innovative Medicines Initiative (IMI)2 PERISCOPE consortium (51, 52) and was approved by the Medical Research Ethics Committees United (MEC-U, NL60807.100.17-R17.039) and registered at the EU Clinical trial registry (EudraCT number 2016-003678-42).

### Immunophenotypic studies

Samples were processed within 4 hours (h) after collection, according to the EuroFlow bulk lysis and sample preparation and staining standard operating procedures (SOP) (52, 53) for surface membrane (Sm) only and Sm plus cytoplasmic (Cy) labeling of 10^7^ cells, employing the antibodies (**Supplementary Table 1**) and antibody combination depicted in **Table 1** and **Supplementary Tables 2, 3**. Protocols are described in detail in the Supplementary Methods section and on the EuroFlow website (www.EuroFlow.org ).

**Table 1:** Antibody combinations used to stain peripheral blood for the selection of the best marker combination for identification of the different innate myeloid cell populations, and overview of the distinct versions evaluated during the multiple design cycles (four rounds) of the EuroFlow innate myeloid cell (IMC) flow cytometry tubes.

	BV421	OC515/BV510	BV605	BV650	BV711	BV786	FITC/BB515	PerCP Cy5.5	PE	PE CF594	PE Cy7	APC	AF700	APC H7/ APC C750	Samples evaluated (n=)
**Backbone**	–	CD45 OC515	CD62L	–	HLA-DR	CD16	–	CD36	Slan	–	–	CD300e	–	CD14 APC H7	–
**pDC**	–	CD45 OC515	–	–	HLA-DR	CD16	CD123 FITC	CD14	CD303	–	CD19	CD304	–	CD300e APC C750	5
	CD141	CD45 OC515	CD62L	–	HLA-DR	CD16	CD1c BB515	CD36	Slan	–	CD33	CD300e + CD304	–	CD14 APC H7	9
	CD141	CD45 OC515	CD62L	–	HLA-DR	CD16	CD1c BB515	CD36	Slan	–	CD33	CD300e + CD303	–	CD14 APC H7	4
**myDC**	CD141	CD5 BV510	CD19	CD11c	HLA-DR	CD16	CD1c BB515	–	–	–	CD33	CD300e + CD303	CD45	CD14 APC H7	5
**Immature neutrophils**	–	CD15 BV510	CD62L	–	HLA-DR	CD16	CD66b BB515	CD64	CD244	CD11b	CD33	CD300e	CD45	CD14 APCH7	5
**M-MDSC**	CD32	CD15 BV510	CD192	CD206	HLA-DR	CD16	–	CD36	CD124	CD11b	CD33	CD300e	CD45	CD14 APC H7	5
	CD32	CD15 BV510	CD192	CD206	HLA-DR	CD16	cyS100A9 FITC	CD36	CD124	CD11b	CD33	CD300e	CD45	CD14 APC H7	3
**Axl^+^ DCs &** **preDCs**	CD141	CD45 OC515	CD36	CD25	HLA-DR	CD33	CD100 FITC	CD1c	Axl	CD34	CD5	CD300e + CD303	CD16	CD14 APC H7	5
**Version 1**	CD141	CD45 OC515	CD62L	–	HLA-DR	CD16	CD1c BB515	CD36	Slan	–	CD33	CD300e + CD303	–	CD14 APC H7	11
**Version 2**	CD141	CD45 OC515	CD62L	–	HLA-DR	CD16	CD1c BB515	CD36	Slan + FcεRI	–	CD33	CD300e + CD303	–	CD14 APC H7	12
**Version 3: 11-color version**	CD141	–	CD62L	–	HLA-DR	CD16	CD1c BB515	CD36	Slan + FcεRI	–	CD33	CD300e + CD303	CD45	CD14 APC H7	121
**Version 4:** **14-color version**	CD141	CD5 BV510	CD192	CD62L	HLA-DR	CD16	CD1c BB515	CD36	Slan + FcεRI	CD34	CD33	CD300e + CD303	CD45	CD14 APC H7	73

AF700, Alexa Fluor 700; APC, allophycocyanin; BB, Brilliant Blue; BV, Brilliant Violet, CF, Cyanin-based Fluorescent dye; Cy7, Cyanin7; FITC, Fluorescein isothiocyanate; H7, Hilite7; PE, Phycoerythrin; PerCP Cy5.5, Peridinin-chlorophyll-protein-cyanin 5.5; M-MDSC, Monocytic myeloid-derived suppressor cells; OC, Orange Cytognos; cy, cytoplasmic.

Stained cells were stored at 4°C and measured within 1h by FCM. Absolute counts were assessed employing a double platform method based on quantitation of nucleated cells obtained in the Sysmex XP-300 automated hematological analyzer (Sysmex Europe GmbH, Norderstedt, Germany).

### *In vitro* activation assay of monocytes and DCs

Short-term *in vitro* activation assays were performed using sodium heparin anti-coagulated PB, as described elsewhere (53). Briefly, 500 μl of PB diluted 1/1 (vol/vol) with RPMI 1640 medium (Sigma-Aldrich, Zwijndrecht, The Netherlands) were incubated for 6h at 37°C in a sterile environment containing 5% CO_2_ in the presence of 100 ng/ml of lipopolysaccharide (LPS) (Sigma-Aldrich). For those experiments in which intracellular detection of cytokines was performed, 10 μg/ml of Brefeldin A (Sigma-Aldrich) was added to block cytokine secretion. For each condition, an unstimulated aliquot of the same sample was processed in parallel in an identical way. Stimulated PB samples were then stained with a panel of monoclonal antibodies (MoAb) (**Supplementary Table 2**) using the EuroFlow bulk lysis and sample preparation and staining SOPs (www.EuroFlow.org) as previously described (54).

### Sample acquisition and analysis

For each sample evaluated, 2.5 to 5 x 10^6^ cells were measured using LSR Fortessa (Becton Dickinson Biosciences (BD), San José, CA) instruments equipped with 4 lasers (405nm, 488nm, 561nm and 640nm) or a 3-laser (405nm, 488nm, 640nm) Aurora (Cytek, Fremont, CA) instrument. BD Fortessa flow cytometers were set-up at each center according to the EuroFlow guidelines (www.EuroFlow.org) and calibrated daily by use of BD™ Setup and Tracking (CS&T) beads (BD Biosciences), their performance being checked daily by acquisition of SPHERO™ Rainbow calibration particles (Cytognos S.L., Salamanca, Spain). Calibration and daily quality control of the Aurora flow cytometer was performed according to the SOP recommended by the manufacturer. For data analysis, the Infinicyt™ software (version 2.0.2.d.000; Cytognos S.L., Salamanca, Spain) was used. Gates were defined based on internal negative and fluorescence-minus-one (FMO) controls, for general population identification and immunophenotypic characterization, respectively.

### Antibody evaluation and selection for the EuroFlow IMC tubes

To design accurate and reproducible antibody combinations for IMC detection in PB, 62 antibodies against 44 proteins were stepwise evaluated in several rounds of EuroFlow-based design–testing–evaluation–redesign (**Table 1**, **Supplementary Table 1**). In a first step, 8 antibodies were used as backbone to accurately identify the major monocytic populations (CD14, CD16, CD45, CD300e, HLA-DR) and their subsets (CD36, CD62L and Slan) (**Table 1**) (9, 37, 39). Selection of different reagents was carried out for each target antigen, based on discrimination between positive and negative reference populations, employing stain index values [calculated as (MFI _PRP_ – MFI _NRP_)/2 x rSD_NRP_; where MFI, median fluorescence intensity; PRP, positive reference population; NRP, negative reference population; rSD, robust standard deviation], as previously described (55).

In a subsequent step, selection of the minimum set of the most informative markers for identification of additional subsets of IMC was performed per cell population, e.g., pDCs, myDCs, Axl^+^ DCs, CD100^+^ preDCs, myeloid-derived suppressor cells (MDSCs) (**Table 1**), using counter-staining with the backbone markers for the major population identification (CD14, CD16, CD45, CD300e, HLA-DR). Selection of individual markers and marker combinations was based on specificity, redundancy, population discrimination and lack of cross-contamination by other cell subsets, as assessed by principal component analysis (PCA) and canonical multivariate analysis (CA) using Infinicyt™ (56). For Axl^+^ DCs, accuracy of the set of markers used for their identification was further validated (**Supplementary Table 2**), employing *in vitro* stimulation of PB samples (n=5).

Based on the above strategies, a first version of the IMC tube was designed (version 1; **Table 1**), further modified to include the anti-FcεRI antibody (version 2; **Table 1**), for better identification of basophils and further subsetting of cMos (36). Subsequently, to improve the discrimination of leukocytes from debris and platelets, and better identify immature neutrophils, the CD45 antibody reagent was replaced with a brighter conjugate in the 11-color version of the tube (version 3; **Table 1**). At a later stage, an extended 14-color version (version 4, **Table 1**) was designed, which also included i) CD5 for further subsetting of CD1c^+^ CD14^-^ myDCs; ii) CD34 for identification of hematopoietic precursor cells (HPCs) and CD100^+^ CD34^int^ pre-DCs; and iii) CD192 for identification of M-MDSCs. Additionally, the fluorochrome conjugated to CD62L was changed to minimize its spread on the FcεRI channel, as required for clear cut subsetting of cMos (**Table 1**).

### Technical performance of the EuroFlow IMC tubes

The technical performance of the EuroFlow IMC tube was evaluated in a Fortessa X20 (BD) for different anti-coagulants (EDTA *vs.* sodium heparin) (assessed in 7 paired PB samples stained with versions 2 (n=3) and 3 (n=4) of the IMC tube; **Table 1**), immediate *vs.* delayed (storage at RT for 6h, 12h and 24h) (n=3; version 3) and fresh *vs.* frozen (n=3; version 4) staining of (EDTA anti-coagulated) PB samples. To compare the performance of the EuroFlow IMC tube in different instruments (i.e., conventional *vs.* spectral flow cytometers), PB samples from 5 donors were stained with version 4 of the tube (**Table 1**) and measured in parallel in a 4-laser Fortessa X20 (BD) conventional flow cytometer (405nm, 488nm, 561nm and 640nm lasers) and a 3-laser Aurora (Cytek) (405nm, 488nm, 640nm) spectral flow cytometer.

### Intra- and inter-assay reproducibility

Intra-assay variation of the EuroFlow IMC tube, expressed by the intra-assay coefficient of variation (%CV) was determined in duplicates of 5 EDTA-anticoagulated PB samples, processed in parallel (version 3; **Table 1**) and measured in a Fortessa X20 (BD) flow cytometer. In addition, inter-center reproducibility was also evaluated *via* analysis of PB samples from HD locally collected, processed (version 3; **Table 1**) and measured at 4 centers: Leiden University Medical Center (LUMC) (n=9), University of Salamanca (USAL) (n=5), National Institute for Public Health and the Environment (RIVM) (n=3), and University of Turku (UTU) (n=4), using five different instruments (2 LSR Fortessa and 3 Fortessa X20). For this purpose, the %CV of the median fluorescence intensity (MFI) obtained for each marker in pre-determined positive reference cell populations was first calculated per center (intra-center variation), and the technical variability between centers (inter-center variation) estimated based on the median MFI of each marker per center.

### Reproducibility of manual data analysis

To evaluate the inter-operator reproducibility of manual analysis, flow cytometry standard (.FCS) sample files from 6 adult HD (stained with version 3 of the EuroFlow IMC tube) were independently analyzed in parallel by an experienced (expert 1 – E1) and a novice (expert 2 – E2) flow cytometrist. Intra-operator variability was assessed for E1, who analyzed the files twice within a time lapse of ≥2 months.

### Database construction for automated data analysis

For construction of the database for automated analysis of the 11-color version of the EuroFlow IMC tube (version 3, **Table 1**), 18 PB samples from healthy adults were processed and measured in Fortessa X20 and LSR Fortessa instruments, at the four different sites mentioned above, within the framework of the Horizon 2020/IMI multicenter PERISCOPE consortium (http://periscope-project.eu/): LUMC (n=5), USAL (n=8), RIVM (n=2), and UTU (n=3). Flow cytometry data files from those 18 samples that fulfilled all the selection criteria (described in detail in Supplementary Methods) were then merged into a single data file used as database tool, implemented in Infinicyt™ (Cytognos) (57). For validation of the database *vs.* manual analysis performed by an experienced flow cytometrist (E1), a second set of PB samples from HD (n=6), processed and acquired at LUMC (n=3) and USAL (n=3), was prospectively used. For these samples, analysis was repeated at two different timepoints set ≥2 months apart from each other.

## Results

### Selection of markers for identification of dendritic cell populations

**Figure 1:**
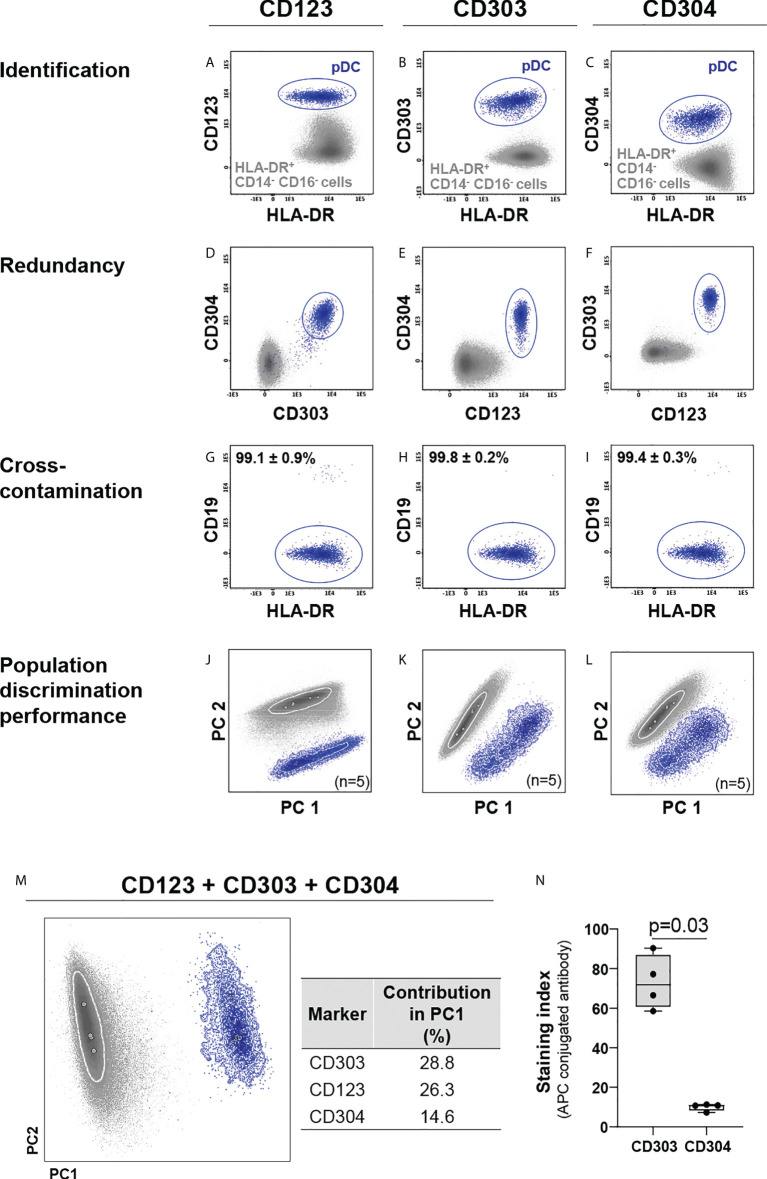
Comparison of the performance of CD123, CD303 and CD304 for identification of plasmacytoid dendritic cells (pDCs). CD123, CD303 and CD304 were individually employed for identification of pDCs **(A–C)** within the HLA-DR+ CD14- CD16- cell population. Redundancy of each marker vs. the other two **(D–F)** and cross-contamination of pDCs with other cells **(G–I)**, were evaluated. Marker performance for discrimination of pDCs vs. other HLA-DR+ CD14- CD16- cells was determined using principal component (PC) analysis (PCA) in the context of cross-staining with CD14, CD16, CD45, CD300e, HLA-DR for the individual markers **(J–L)** and in the combination of all markers **(M)**. The contribution of CD123, CD304 and CD303 to the separation of pDCs vs. other HLA-DR+ CD14- CD16- cells is depicted in the table. **(N)** exhibits the staining index of allophycocyanin (APC)-conjugated CD303 and CD304 reagents. To test differences, the Mann-Whitney test was used. Solid circles in all PCA plots represent median values for the parameters evaluated and dotted lines depict the first standard deviation for each population identified. pDC, plasmacytoid dendritic cells; PC, principal component; APC, allophycocyanin.

Based on earlier work (9, 37–39), a set of eight markers (CD14, CD16, CD36, CD45, CD62L, CD300e, HLA-DR and Slan) that allows for identification and subsetting of monocytic cells, was pre-selected as backbone (**Table 1**), based on antibody clones that had previously shown to perform well technically (**Supplementary Table 1**) (9, 37, 39, 58). Three markers (CD123, CD303 and CD304) were evaluated for the specific identification of pDCs (25, 27, 31) in combination with the backbone combination required for identification of the major populations of monocytes and granulocytes (CD14, CD16, CD45, CD300e and HLA-DR). All three markers individually allowed clear identification of pDCs within the HLA-DR^+^/CD14^-^/CD16^-^ cell compartment (**Figures 1A–F**). However, whereas the CD303 and CD304 expression was highly specific for pDCs, CD123 was also present in other cell populations (**Figures 1A–C**), such as HPCs, myDC and some B-cell (sub)populations (40, 59). While no significant differences were observed for population purity (**Figures 1G–I**), multivariate analysis (PCA) showed the highest discrimination power for CD303 (28.8%) *vs.* CD123 (26.3%) and CD304 (14.6%) (**Figures 1J–M**), even when CD303 and CD304 antibody reagents conjugated with the same fluorochrome were compared (p=0.03, **Figure 1N**). Importantly, since CD303 and the backbone marker CD300e are not expressed on the same cells (i.e., monocytes/myDCs *vs.* pDCs, respectively) (**Supplementary Figure 1**), both antibodies could be used in the later versions of the antibody combination with the same fluorochrome.

**Figure 2:**
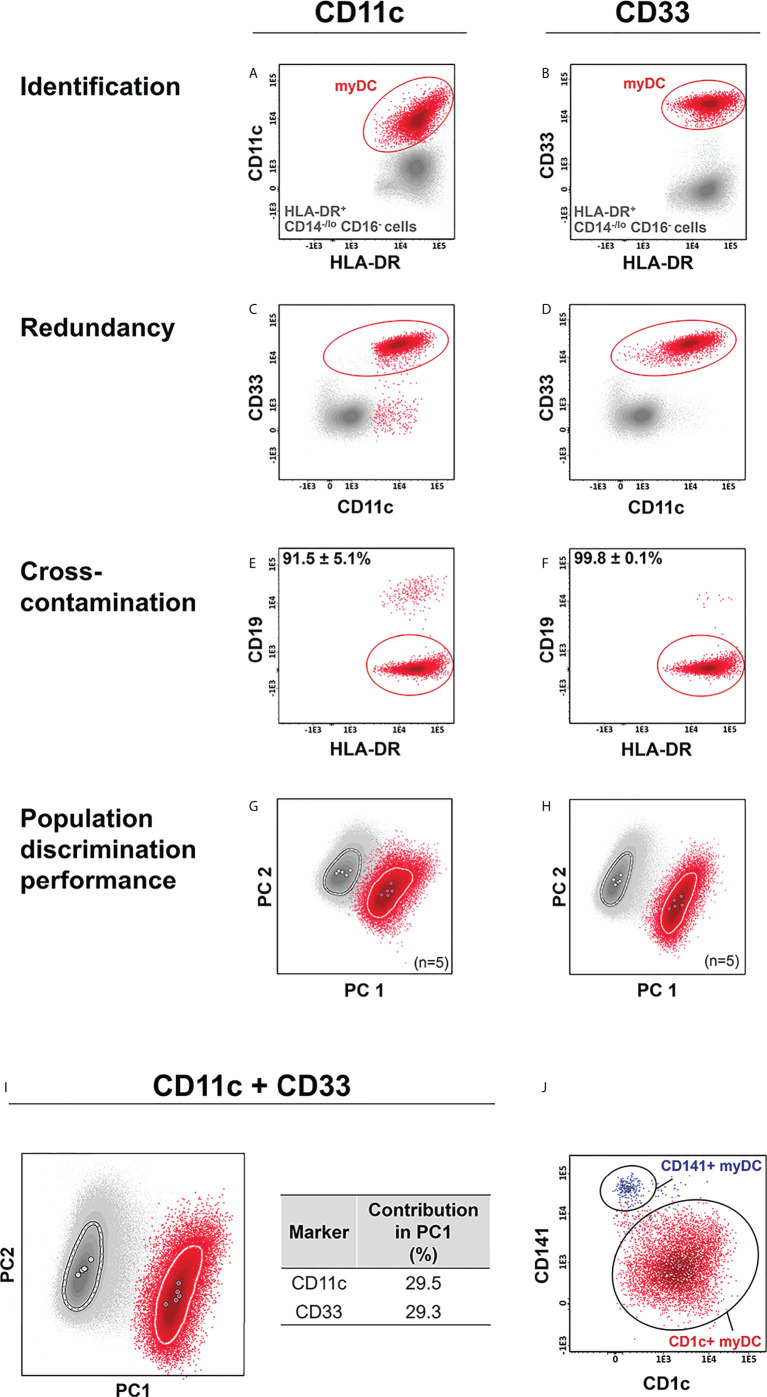
Comparison of the performance of CD11c *vs.* CD33 for identification of myeloid dendritic cells (myDCs). CD11c and CD33 were individually employed for identification of myDCs **(A, B)** within the HLA-DR^+^ CD14^-/lo^, CD16^-^ cell population. Redundancy of each marker *vs.* the other (**C, D**), and cross-contamination with other cells **(E, F)**, was evaluated. Marker performance for identification of myDCs vs. other HLA-DR^+^ CD14^-/lo^, CD16^-^ cells was determined using principal component (PC) analysis (PCA) in the context of cross-staining with CD14, CD16, CD45, HLA-DR for the individual markers **(G, H)** and in the combination of all markers **(I)**. The contribution of CD11c and CD33 for the discrimination of the two populations is depicted in the table. **(J)** shows a representative example of CD1c^+^ myDCs and CD141^+^ myDCs subsetting within the myDC population, previously identified based on expression of CD33. Solid circles in all principal component plots represent median values for the parameters evaluated and dotted lines depict the first standard deviation for each population identified. myDC, myeloid dendritic cells; PC, principal component.

For myDCs, CD11c and CD33 were selected to be tested (38, 41, 42) in combination with the backbone markers. As expected, both markers allowed accurate identification of myDCs within the HLA-DR^+^/CD14^-/lo^/CD16^-^ cell compartment (**Figures 2A**–**D**), with a similar discrimination power (**Figures 2G–I**). Nevertheless, CD33 exhibited higher specificity than CD11c (purity of 99.8% ± 0.1% *vs.* 91.5% ± 5.1%, respectively) (**Figures 2E, F**) and was, therefore selected for identification of myDCs. Further discrimination among myDCs between conventional type 1 (cDC1) and 2 (cDC2) DCs, based on CD141 (BDCA3) and CD1c (BDCA-1) was then successfully evaluated (**Figure 2J**) (27, 60–62). Additional subsetting of CD1c^+^ myDCs has been recently reported based on the low *vs.* absent expression of CD14 with functional implications (the former show a more inflammatory gene expression profile) (40). Likewise, low *vs.* high expression of CD5 provided the ability for further functional subclassification of CD1c^+^ myDC (42), and this marker was therefore included in the extended version 4 of the IMC tube (**Table 1**), which allowed for unequivocal identification of three subsets of CD1c^+^ myDC (CD14^lo^, CD14^-^ CD5^-^ and CD14^-^ CD5^+^) (**Supplementary Figure 2**).

### Detection of CD100^+^ preDCs in PB does not need a CD100 antibody

Even though the definition of the myDC precursor is still elusive, a PB population identified based on a CD34^int^ CD100^hi^ immunophenotype (**Supplementary Figure 3**), with the ability to differentiate to both CD1c^+^ and CD141^+^ myDC, has been described in PB (40). PCA performed on PB cells stained with CD34 and CD100 in combination with CD14, CD16, CD33, CD45, CD300e, CD303 and HLA-DR exhibited a clear separation between CD34^+^ HPC and CD100^+^ DC precursors mostly due to their different pattern of expression of HLA-DR^hi^ and CD34^int^ (**Supplementary Figure 3B**), showing that CD100 is not critically required, as a similar discrimination power was observed when CD100 was excluded (**Supplementary Figures 3C-D**). Based on these results, CD34, but not CD100, was included in the extended 14-color version 4 of the IMC antibody panel (**Table 1**).

### Detection of Axl^+^ DCs does not need an Axl antibody

**Figure 3:**
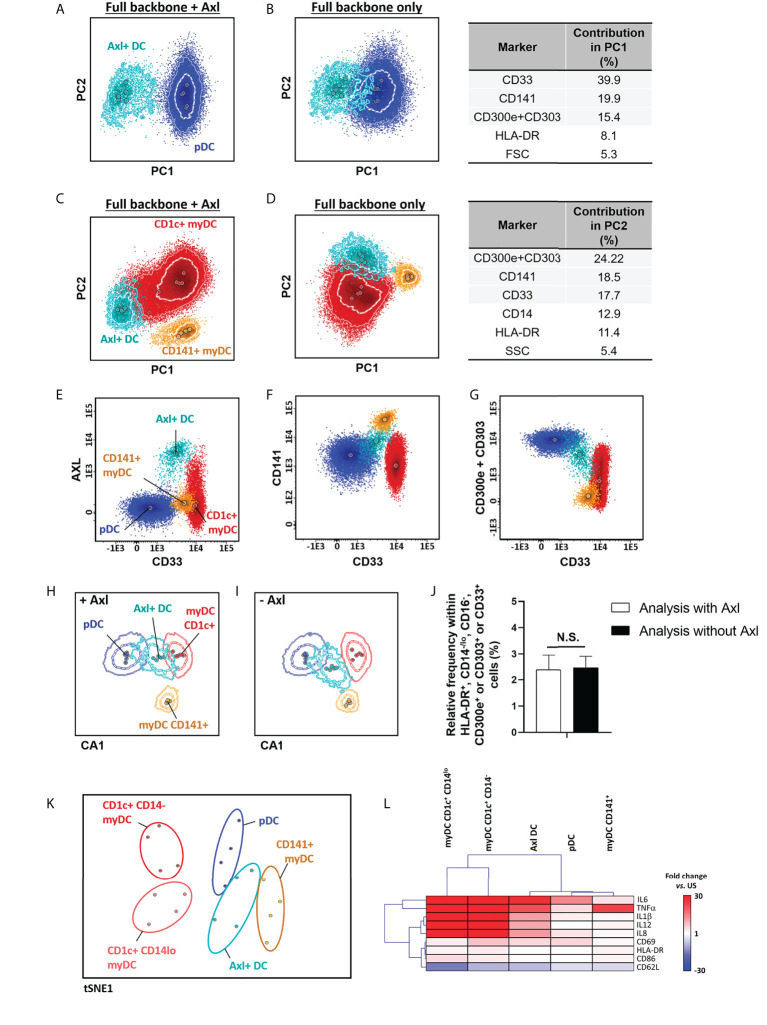
Identification and functional characterization of Axl^+^ dendritic cells (DCs) *vs.* other DC populations. Identification of Axl^+^ DCs within the HLA-DR^+^ CD14^-/lo^, CD16^-^, CD300e^+^ or CD303^+^ or CD33^+^ cell population *vs.* plasmacytoid **(A, B)** and myeloid **(C, D)** DCs, in the context of staining with CD1c, CD14, CD16, CD33, CD45, CD141, CD300e+CD303 (i.e. full backbone) alone **(B, D)** or in combination with Axl **(A, C)** (n=5) is shown. Performance of the full backbone for discrimination of Axl^+^ DCs *vs.* other DCs, and the relative contribution of the most informative markers (>5%) for the separation between populations is depicted in **(B, D)**. Expression patterns of the minimum set of markers required for identification of Axl+ DCs are shown in panels **(E–G)**, respectively. **(H, I)** represent the canonical multivariate analysis (CA) for overall discrimination of DC populations (n=5 donors). Relative frequency of Axl^+^ DCs after staining with CD33, CD141 and CD300e+CD303 with and without Axl, is depicted in **(J)** (n=5). The t-distributed stochastic neighbor embedding (t-SNE) plot in **(K)** depicts the overall expression of activation- and maturation-related markers which showed statistically significantly different expression patterns at steady-state and in response to stimulation with LPS (TNFα, IL1β, IL6, IL8, IL12, CD33, CD62L, CD63, CD69, CD83, CD86 and HLA-DR) between the distinct DC populations (n=4 donors). **(L)** shows a hierarchical clustering analysis of the response to stimulation with LPS (given as fold change *vs.* steady-state) of the distinct DC populations identified employing the backbone set of markers. Statistical differences were evaluated employing Kruskall-Wallis and Wilcoxon tests with a false discovery rate (FDR) of 5% to correct for multiple comparisons, to compare between populations and steady-state *vs.* stimulation, respectively. Solid circles in all principal component, canonical analysis and tSNE plots represent median values for the parameters evaluated, inner dotted and outer solid lines depict the first standard and second standard deviations for each population identified. pDC, plasmacytoid dendritic cells; myDC, myeloid dendritic cells; PC, principal component; CA, canonical multivariate analysis; N.S., not statistically significant (p value>0.05); tSNE, t-distributed stochastic neighbor embedding; US, unstimulated.

In 2017, Villani *et al.* (40) described a new population of DCs that overlaps with pDCs, when classical identification markers are used, but that could be accurately discriminated based on the expression of Axl. In order to identify Axl^+^ DC, Axl was combined with CD1c, CD14, CD16, CD33, CD45, CD141, CD303, CD300e and HLA-DR. Overall, inclusion of Axl in the antibody combination proved not to be critically required for identification of this DC population, as the expanded backbone combination allowed for the separation of the Axl^+^ DCs from pDCs and myDC populations based on its unique pattern of expression of CD33^lo^, CD141^+^ and CD303^lo^ (**Figures 3A–G**). This was further confirmed by multivariate analysis, which revealed similar population discrimination patterns, independently of the presence or absence of Axl (**Figures 3H,I**), associated with similar Axl^+^ DC counts (**Figure 3J**). Axl^+^ DCs have been reported to display a mixed gene expression signature between myDC and pDC, with shared immunophenotypic features with pDCs (e.g., CD123 and CD303 expression) and functional characteristics of myDCs (e.g., response to LPS) (40). Therefore, we further validated the functional identity of the Axl^+^ DC population, identified based on the restricted set of markers selected for evaluation of DC populations, (**Table 1**; **Supplementary Table 2**). Our results showed that in unstimulated samples, expression of CD11b was restricted to myDCs, whereas CD33 was also (dimly) expressed on Axl^+^ DCs, but not on pDCs. In turn, steady-state Axl^+^ DCs displayed a higher frequency of pro-inflammatory cytokine producing cells *vs.* myDCs CD1c^+^ CD14^-^ (p<0.02 for IL1β and IL12) and pDCs (p<0.003 for IL1β and IL8) (**Supplementary Figures 4, 5**). Upon exposure to LPS, CD1c^+^ myDCs populations displayed a strong response to LPS, while pDCs and CD141^+^ myDCs were mostly unresponsive, Axl^+^ DCs exhibited an overall intermediate activation pattern, associated with a unique profile for those markers that showed significant differences in steady-state and/or in LPS-stimulated samples (CD33, CD62L, CD63, CD69, CD83, CD86, IL1β, IL6, IL8, IL12 and TNFα) (**Figures 3K, L**).

### Selection of markers for identification of immature *vs.* mature neutrophils

**Figure 4:**
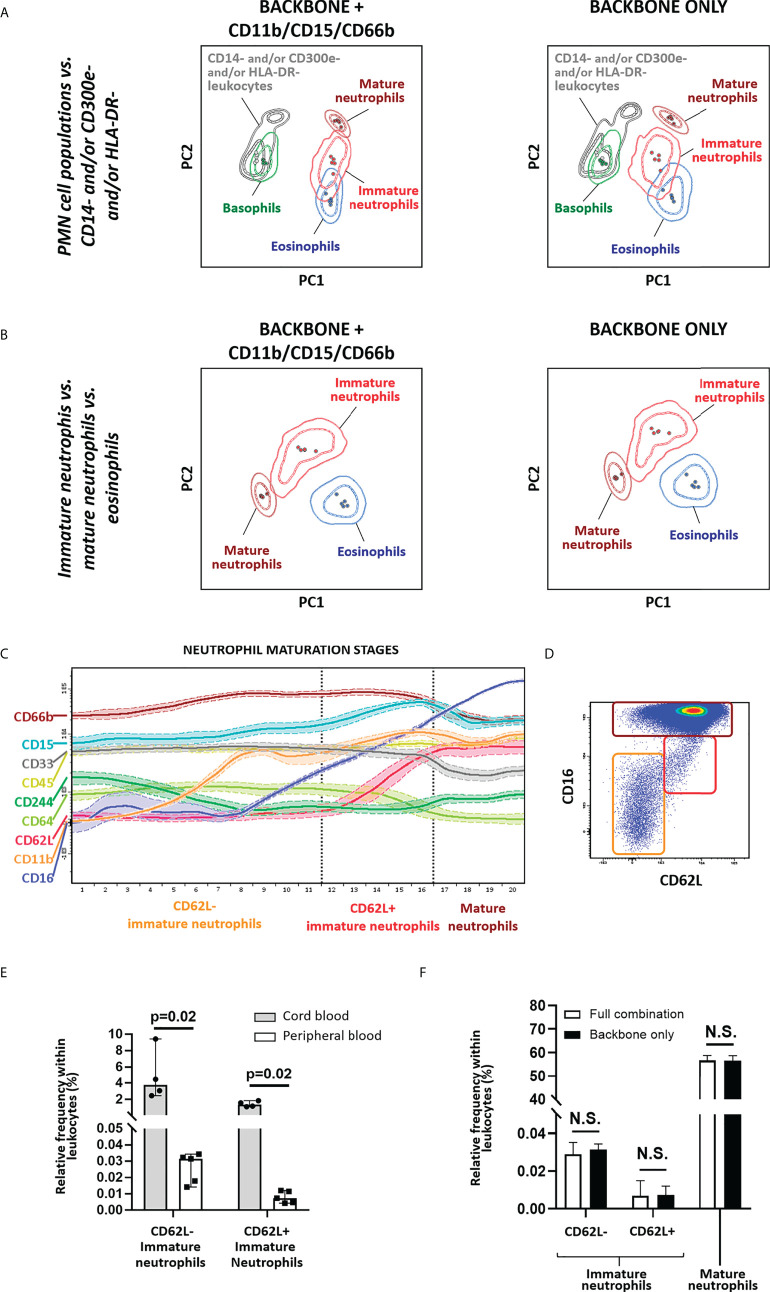
Identification of immature neutrophils. Principal component analysis (PCA) plots depicting the overall performance of backbone markers for general monocytic and dendritic cell identification (CD14, CD16, CD33, CD45, CD62L, CD300e, HLA-DR) in combination with size (FSC) and internal complexity (SSC) *vs.* same combination plus CD11b, CD15, CD66b, for identification of polymorphonuclear (PMN) cell populations (basophils, eosinophils and neutrophils) **(A)** and for identification of immature *vs.* mature neutrophils and eosinophils, within the SSC^hi^ cell compartment **(B)**. Wanderlust plot exhibiting the modulation of markers related with neutrophil maturation is depicted in **(C)**, whereas a dot plot depicting the different neutrophil populations identified employing the backbone markers is shown in **(D)**. **(E)** depicts the relative frequency of immature neutrophils in cord blood (CB) (n=4) *vs.* adult peripheral blood (PB) (n=5), whereas **(F)** displays the impact on the relative frequency of the populations with inclusion of CD11b, CD15 and CD66b *vs.* backbone combination only. Statistical differences were evaluated employing Wilcoxon and Mann-Whitney tests, to compare between gating strategies and CB *vs.* PB, respectively. Solid circles in the PCA plot represent median values for the parameters evaluated, inner dotted and outer solid lines depict the first standard and second standard deviations for each population identified. Expression in the Wanderlust plot is reported as median fluorescence intensity (solid line) and one standard deviation (dotted line). Bars on graphs depict the median and 95% confidence interval. PMN, polymorphonuclear; PC, principal component. N.S., not statistically significant (p value>0.05).

In order to determine whether additional markers are required for accurate identification of immature *vs.* mature neutrophils, PCA-based evaluation of the performance of the IMC tube extended backbone (i.e., backbone markers plus the markers required for identification of DCs) *vs.* the extended backbone plus CD11b, CD15 and CD66b, for identification of different polymorphonuclear (PMN) cells, including immature neutrophils, was performed. Of note, combined usage of cell size (forward scatter – FSC-) and internal complexity (side scatter – SSC-) plus CD45 allowed for clear separation of granulocyte and lymphocyte populations. Likewise, the SSC^hi^ CD16^-^ CD33^lo^ CD62L^hi^, SSC^lo^ CD16^-^ CD33^hi^ CD62L^hi^ and SSC^hi^ CD16^hi^ CD33^lo^ CD62L^hi^ phenotypic profiles allowed clear discrimination among eosinophils, basophils, and mature neutrophils, respectively, as well as their distinction from SSC^int^ CD16^-/lo^ CD33^+^ CD62L^-/lo^ immature neutrophils, with no clear added value of the other myeloid markers evaluated (**Figures 4A,B**). This was also confirmed by the expression pattern of markers known to be associated and/or modulated during neutrophil maturation (**Figure 4C**), as cells identified based on an HLA-DR^-^ CD14^-^ CD16^-/lo^ CD33^+^ CD45^lo^ CD300e^-^ phenotype in fact correspond to immature (CD11b^-/+^, CD15^+^, CD66b^+^, CD244^-/lo^) neutrophils. Interestingly, neutrophils could be further subclassified based on expression of CD16 and CD62L (**Figure 4D**) as mature neutrophils (CD16^hi^ CD62L^+^), a phenotype previously reported to be associated with segmented neutrophils (63), immature neutrophils CD16^lo^ CD62L^+^, compatible with band neutrophils (63), and other, even more immature subsets of CD16^-/lo^ CD62L^-^ neutrophils, that might include an admixture of promyelocytes (CD11b^-^), myelocytes (CD11b^+^) and metamyelocytes (CD16^lo/+^) (**Figures 4C, D**) (64). As expected (65), significantly higher frequencies of immature neutrophils were observed in CB samples *vs.* adult PB with the extended backbone (**Figure 4E**), with similar immature neutrophil counts in the presence *vs.* absence of additional neutrophil-associated markers (**Figure 4F**).

### Selection of markers for identification of monocytic myeloid-derived suppressor cells

Monocytic M-MDSCs have been classically identified as CD14^+^ CD11b^+^ (or CD33^+^) CD15^−^ and HLA-DR^−/lo^ cells (32). This combination relies on the expression of HLA-DR as the discriminating marker *vs.* cMos, which requires FMO or internal negative controls for accurate identification of this cell population. To specifically identify markers that would allow for an improved identification of M-MDSCs, we evaluated the pattern of expression of monocyte and M-MDSC-related markers on cMos *vs.* CD14^+^ HLA-DR^-/lo^ cells from CB and/or adult PB samples (**Supplementary Figure 6A**). Our results confirmed the absence of CD15 together with expression of CD11b on CD14^+^ HLA-DR^-/lo^ M-MDSCs, and showed significant (p=0.03) up-regulation of CD16 and down-regulation of CD123 and CD192 on CD14^+^/HLA-DR^-/lo^ cells *vs.* cMos (**Supplementary Figures 6B, C**). PCA revealed that only CD16, HLA-DR and CD192 had significant (independent) impact on the discrimination between the two populations (**Supplementary Figure 6D**), with addition of CD123 having negligible value for identification and quantification of the population (**Supplementary Figure 6E**). When comparing the frequency of M-MDSC in CB *vs.* adult PB, defined based on a CD14^+^ HLA-DR^-/lo^ or CD14^+^ HLA-DR^-/lo^ CD192^-/lo^ phenotype, lower frequencies were overall observed with the latter, more stringent, criteria (**Supplementary Figure 6F**). Importantly, statistically significantly higher frequencies of M-MDSCs in CB *vs.* adult PB were only observed when the CD14^+^ HLA-DR^-/lo^ CD192^-/lo^ criteria was used (**Supplementary Figure 6F**), suggesting that the addition of CD192 could allow for a more accurate identification of M-MDSCs.

### Comparison of the performance of versions 3 and 4 of the EuroFlow IMC tubes

**Figure 5:**
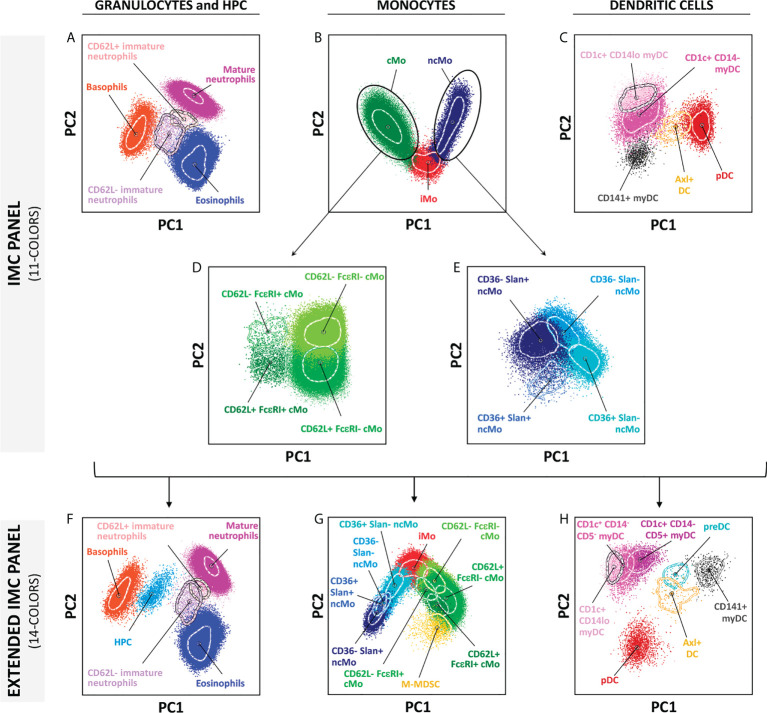
Multidimensional representation (principal component analysis – PCA) of the cell populations identified in one representative adult blood sample, employing the two versions of the innate myeloid cell panel. **(A–E)** show the 19 populations identified using version 3 (11-colors) antibody combination, whereas **(F–H)** depict the 23 populations identified in the extended version of the panel (version 4; 14-colors). Solid circles represent median values for the parameters evaluated, inner dotted lines depict the first standard deviations for each population identified. PC, principal component; cMo, classical monocytes; iMo, intermediate monocytes; ncMo, non-classical monocytes; myDC, myeloid dendritic cells; pDC, plasmacytoid dendritic cells; M-MDSC, monocytic-myeloid derived suppressor cells; HPC, hematopoietic precursor cells; preDC, CD100^+^ dendritic cell precursors.

As described above, two different versions of EuroFlow IMC antibody combinations were designed, which included a more restricted 11-color combination (version 3, **Table 1**) suitable for *in vitro* diagnostics (CE-IVD)-certified instruments (e.g., BD FACSLyric™ flow cytometer), and an extended 14-color version (version 4, **Table 1**) for additional identification of other minor (and less frequently reported) populations, such as HPCs, M-MDSCs, preDCs, and further subsetting of CD1c^+^ CD14^-^ myDCs into their CD5^-^ and CD5^+^ subsets (**Figure 5** and **Supplementary Figure 7**). Both versions of the EuroFlow IMC tube allow for the identification of 5 subsets of granulocytes (basophils, eosinophils, mature neutrophils, immature neutrophils CD62L^-^ and CD62L^+^), 9 populations of monocytes (4 subpopulations of cMos defined based on CD62L^+^ FcϵRI^+^, CD62L^+^ FcϵRI^-^, CD62L^-^ FcϵRI^-^ and CD62L^-^ FcϵRI^+^ expression patterns, iMo and 4 subsets of ncMos identified as CD36^+^ Slan^+^, CD36^+^ Slan^-^, CD36^-^ Slan^-^ and CD36^-^ Slan^+^) and 5 populations of DC (CD1c^+^ CD14^lo^, CD1c^+^ CD14^-^ and CD141^+^ myDCs, pDCs and Axl^+^ DCs) (**Figure 5**; **Supplementary Figure 7** and **Supplementary Table 4**).

### Impact of the anticoagulant, delayed sample preparation and freezing on identification of IMC populations

**Figure 6:**
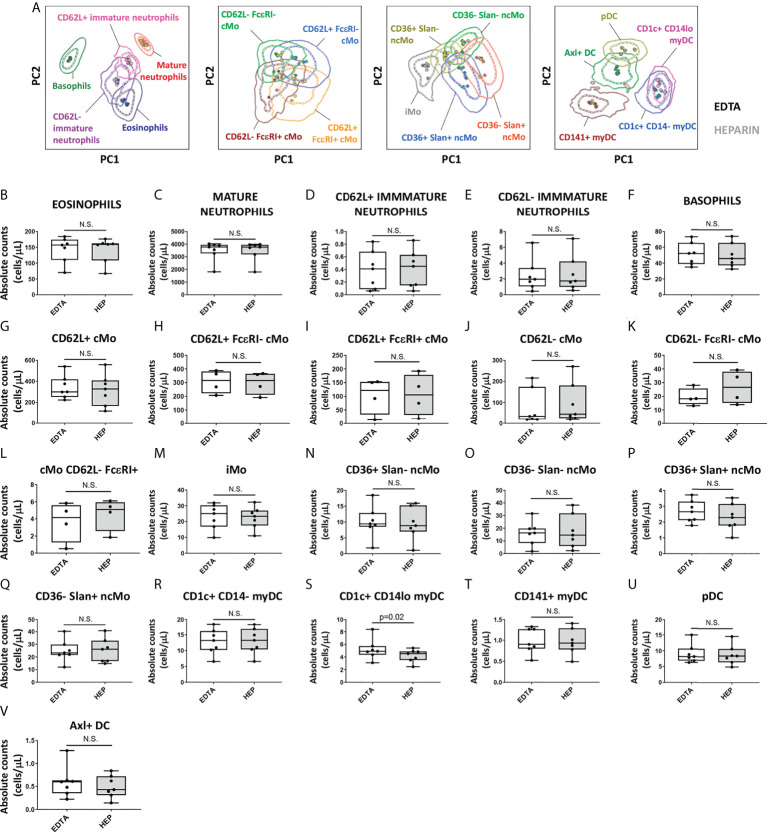
Impact of the anticoagulant on the staining patterns and IMC population (absolute) counts in blood. Peripheral blood samples (n=7) were collected into K3 ethylenediaminetetraacetic acid (EDTA) and sodium heparin (HEP) tubes and stained with versions 2 (n=3) and 3 (n=4) of the EuroFlow immunemonitoring innate myeloid tube. **(A)** depicts principal component analysis (PCA) plots comparing the staining patterns for the populations identified in samples collected in EDTA *vs.* HEP (version 3; n=4). **(B-V)** show the impact of the anticoagulant used for sample collection on the absolute counts of the different IMC populations identified using the EuroFlow innate myeloid cell tube. Statistical differences were evaluated empoying the Wilcoxon test. Solid circles in all PCA plots represent median values for the parameters evaluated in each sample, inner dotted and outer solid lines depict the first standard and second standard deviations for each population identified in the EDTA-anticoagulated samples. cMo, classical monocytes; iMo, intermediate monocytes; ncMo, non-classical monocytes; pDC, plasmacytoid dendritic cells; myDC, myeloid dendritic cells; PC, principal component; EDTA, ethylenediaminetetraacetic acid; HEP, sodium heparin. N.S., not statistically significant (p value>0.05).

Since the performance of the EuroFlow IMC tube was evaluated on PB collected in EDTA and, in some settings, sodium heparin (e.g., for functional assays) is required, which might affect the staining patterns and quantification of IMC populations (66), staining of samples collected with EDTA *vs.* sodium heparin was compared. Except for CD300e that showed lower expression on monocytes from heparin samples (median stain index reduction in heparin *vs.* EDTA of 38.4%; range: 14.2%-71.1%; p=0.02), no significant differences were observed in the stain index of individual markers between samples collected with these two anticoagulants (data not shown). However, despite the lower CD300e expression on heparin-anticoagulated samples, multivariate PCA analyses revealed no significant impact on the overall discrimination of the distinct populations of IMCs (**Figure 6A**). Likewise, no significant differences were observed on the absolute counts of the populations between the two anticoagulants with exception of a lower absolute count of CD1c^+^ CD14^lo^ myDC observed in heparin samples (p=0.02) (**Figures 6B–V**).

**Figure 7:**
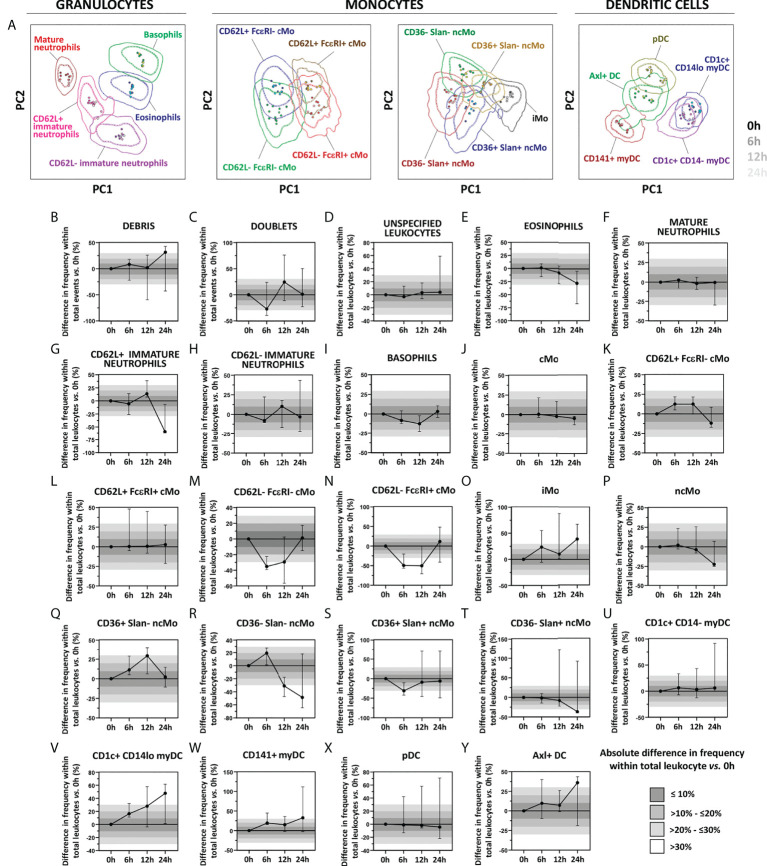
Impact of delayed sample processing on the overall performance of the innate myeloid panel for population identification and quantification of innate myeloid cell (IMC) populations in blood. Principal component analysis (PCA) plots reflecting the impact of sample storage (6h, 12h and 24h, shown as different shades of the population color) on the immunophenotypic patterns vs. samples processed immediately upon collection (0h) are depicted in Panel **A**. Differences in frequency of the IMC populations detected with version 3 of the innate myeloid cell tube as a result of delayed processing vs. freshly stained samples are shown in Panels **B–Y**. Absolute differences vs. 0h staining are depicted with bars of different shades of grey. Solid circles in all PCA plots represent median values for the parameters evaluated in each sample, inner dotted and outer solid lines depict the first standard and second standard deviations for each population identified in 0h condition. Data in the timecourse plot is reported as median and 95% confidence interval. cMo, classical monocytes; iMo, intermediate monocytes; ncMo, non-classical monocytes; pDC, plasmacytoid dendritic cells; myDC, myeloid dendritic cells; PC, principal component.

Regarding immediate *vs.* delayed sample preparation and staining for 6h, 12h and 24h with the EuroFlow IMC tube, similar stain index values were observed for all markers evaluated (data not shown), except for CD16 (median decrease in stain index of 25.3%, 38.5% and 41.9%, respectively) and Slan (median decrease in stain index of 43.2%, 5.4% and 8.1%, respectively), also confirmed by PCA analyses, as all populations evaluated for all timepoints tested clustered within one standard deviation of the 0h staining pattern (**Figure 7A**). Despite no significant impact was detected on the overall discrimination among the different cell populations up to 24h after sample collection, delayed sample preparation was associated with differences *vs.* 0h >10% for 62.5% (15/24) of the populations evaluated (**Figures 7B–Y**). More specifically, one population (4.2%; 1/24) displayed highly variable distribution across the timepoints tested (i.e., cell doublets) (**Figure 7C**), while 33.3% (8/24) and 25% (6/24) of IMC populations showed altered cell counts at >6h and >12h, respectively. Of note, quantification of CD62L^-^ cMo populations was more strongly affected than CD62L^+^ cMo, leading to underestimation of the frequency of the former cMo populations (**Figures 7M, N**). Similarly, CD36^-^ ncMo populations, eosinophils and CD62L^+^ immature neutrophils displayed decreased numbers (*vs.* 0h) when sample preparation was delayed for >12h (**Figures 7E, G, R, T**). Conversely, overestimation of iMos, was observed at all timepoints tested (**Figure 7O**). While pDCs and CD1c^+^ CD14^-^ myDC remained stable up to 24h, delayed sample preparation was generally associated with an overestimation of (minor) DC populations (**Figures 7V, W, Y**).

Analysis of paired freshly processed *vs.* frozen PB mononuclear cells (PBMCs) revealed that, despite freezing induced a significant (>15%) reduction in the MFI of PRP for Slan (-24.8% ± 5.7%), CD192 (-28.4% ± 3.9%), CD5 (-33.0% ± 5.0%) and CD62L (-52.2% ± 10.1%), with the exception of immature neutrophils, which could not be detected in frozen samples, all IMC populations could be identified in both conditions (**Supplementary Figure 8**). Furthermore, the freezing process had a significant impact on the relative frequency of several populations, leading to e.g., overestimation of DCs, CD62L- *vs.* CD62L+ cMos and CD36+ *vs.* CD36- ncMos (**Supplementary Figure 8**).

### Technical performance of the EuroFlow IMC tubes: intra-assay variability, reproducibility in different flow cytometer platforms and multicentric applicability

To determine the assay reproducibility, duplicates of the same EDTA-anticoagulated PB samples (n=5) were stained and measured in the same instrument and analyzed manually by an expert cytometrist. Overall, an average intra-assay %CV of 5.0% ± 4.5% was observed across the 26 populations evaluated, with 80.8% (21/26) of the populations displaying an intra-assay %CV <10% and only CD36^-^ Slan^-^ ncMo exhibiting a median intra-assay %CV >15% (**Supplementary Table 5**).

Comparison of the performance of the EuroFlow IMC tube between different instruments with distinct detector/optical configurations (conventional *vs.* spectral, and 3- *vs.* 4-laser flow cytometers) was evaluated. Overall, a significant correlation (R^2^>0.90; p<0.05) was observed for virtually all (92%; 23/25) IMC populations identified, with no significant differences and a limited bias (absolute mean normalized bias <15%) being detected between instruments. The only exceptions were CD62L^+^ FcεRI^+^ cMos and CD36^+^ Slan^+^ ncMos which were overestimated (bias: +17.5%) and underestimated (bias: -32%) in the data files generated in the Aurora *vs.* Fortessa X20 instruments, respectively (**Table 2**).

**Table 2:** Comparative evaluation of the relative frequency of the innate myeloid cell (IMC) (sub)populations identified with the 14-color version (version 4) of the EuroFlow Innate Myeloid Cell tube in paired samples measured employing two different instruments [Fortessa X20 (BD Biosciences) and Aurora (Cytek)] (n=5).

Populations	Relative frequency from total leukocytes Fortessa X20 (%)[median (min-max)]	Correlation between instruments[R^2^]	Difference in population frequency between instruments [p-value]	MNB[%]
Eosinophils	3.3 (0.9-6.2)	0.987	N.S.	3.1
Neutrophils	49.3 (49.0-60.7)	0.997	N.S.	-2.6
Mature neutrophils	49.3 (49.0-60.6)	0.997	N.S.	-2.6
Immature neutrophils	0.01 (0.01-0.07)	0.997	N.S.	3.5
CD62L+ immature neutrophils	0.009 (0.005-0.07)	0.996	N.S.	7.3
CD62L- immature neutrophils	0.004 (0.003-0.009)	0.995	N.S.	-3.3
Basophils	1.1 (0.5-1.3)	0.998	N.S.	3.1
Monocytes	5.9 (5.3-12.4)	0.998	N.S.	3.9
cMo	5.1 (4.2-10.2)	0.996	N.S.	4.6
CD62L+ FcεRI- cMo	4.7 (3.6-6.5)	0.951	N.S.	1.4
CD62L+ FcεRI+ cMo	0.3 (0.07-2.2)	0.995	N.S.	17.5
CD62L- FcεRI- cMo	0.3 (0.20-1.2)	0.823	N.S.	-4.6
CD62L- FcεRI+ cMo	0.03 (0.007-0.4)	1.000	N.S.	-0.5
iMo	0.2 (0.2-0.4)	0.966	N.S.	2.6
ncMo	0.6 (0.4-1.8)	1.000	N.S.	-2.9
CD36+ Slan- ncMo	0.3 (0.2-0.7)	0.995	N.S.	-1.3
CD36- Slan- ncMo	0.2 (0.1-0.9)	0.998	N.S.	-5.6
CD36+ Slan+ ncMo	0.03 (0.02-0.04)	0.600	N.S.	-32.0
CD36- Slan+ ncMo	0.1 (0.07-0.2)	0.989	N.S.	3.3
CD1c+ myDCs	0.3 (0.2-0.5)	0.974	N.S.	-1.8
CD1c+ CD14- myDC	0.2 (0.2-0.4)	0.944	N.S.	-5.2
CD1c+ CD14lo myDC	0.07 (0.03-0.1)	0.978	N.S.	9.1
CD141+ myDC	0.02 (0.009-0.02)	0.953	N.S.	-0.3
pDC	0.2 (0.1-0.2)	0.951	N.S.	4.5
Axl^+^ DC	0.009 (0.003-0.01)	0.974	N.S.	-0.3
**% of populations with R^2^ ≥ 0.9 and p ≤ 0.05** **or -15% < MNB < + 15%**		**92%** (23/25)	**0%** (0/25)	**92%** (23/25)

For determination of the comparability between samples measured using different types of instruments (conventional vs. spectral cytometers) regarding the relative distribution of the populations, a linear regression was performed to evaluate the direction and strength of the relationship between the two conditions, a Wilcoxon test was performed to compare the differences observed between the two conditions and a Bland-Altman analysis was done in order to determine the potential bias.

MNB, mean normalized bias (calculated as % of difference between the relative frequencies obtained with the Aurora compared to the results obtained with the Fortessa X20); N.S., not significant (p<0.05); cMo, classical monocytes; iMo, intermediate monocytes; ncMo, non-classical monocytes; myDC, myeloid dendritic cells; pDC, plasmacytoid dendritic cells.

**Figure 8:**
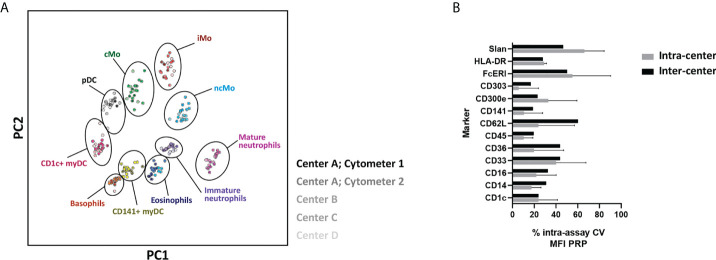
Performance of the EuroFlow immunemonitoring innate myeloid cell (IMC) tube in a multicentric setting. **(A)** Principal component analysis (PCA) plot depicting the staining profile of 21 healthy adult peripheral blood samples processed at 4 centers and measured in 5 different instruments, employing the 11-color version (version 3) of the EuroFlow innate myeloid cell tube. Solid circles, represent the median values of the populations and the shades of color represent different centers and/or instruments. **(B)** depicts the intra-assay % of coefficient of variation for individual markers within centers (intra-center; biological variation) and between centers (inter-center, technical variation). cMo, classical monocytes; iMo, intermediate monocytes; ncMo, non-classical monocytes; pDC, plasmacytoid dendritic cells; myDC, myeloid dendritic cells; PC, principal component; CV, coefficient of variation; MFI PRP, Median Fluorescence Intensity of Positive Reference Population.

To further evaluate the feasibility of using the EuroFlow IMC tube in multicentric settings, 21 samples were locally collected, processed, and measured at 4 distinct facilities (LUMC, USAL, RIVM and UTU) using 5 distinct instruments. PCA revealed fully comparable and reproducible results for all centers/instruments (**Figure 8A**). Furthermore, when comparing the assay %CV for MFI values of predefined positive reference IMC populations (PRP) for the different markers evaluated, the inter-center assay %CV was within the range of the observed biological variability (i.e., intra-assay %CV) within individual centers (**Figure 8B**) (median assay %CV and range of 33.8% [13.9% - 60.3%] *vs.* 30.7% [1.2% – 90.4%], respectively).

### Reproducibility of expert-based manual analysis

Reproducibility of expert-based manual analysis of the EuroFlow IMC tube was evaluated by experienced (E1) and novice (E2) analysts in 6 ,FCS files, stained with version 3 (**Table 1**) of the combination. Overall, a good correlation (R^2^>0.90; p-value <0.05) with a limited bias - absolute mean normalized bias (MNB) <15% - in population counts was observed between the experts (71.4%; 20/28) (**Table 3**). However, a lower correlation and degree of agreement were observed for populations identified based on a limited number of heterogeneously expressed markers (e.g., cMo populations defined based on CD62L and FcεRI expression and ncMo populations, defined based on expression of CD36 and Slan) and infrequent (<0.05% of total leukocytes) IMC populations (e.g., Axl^+^ DCs). To establish the intra-operator variability, expert E1 repeated the analysis of the files with a ≥2-month interval. Of note, even though the overall degree of correlation increased compared to expert E1 *vs.* E2 (significant correlation of 78.6%; 22/28 *vs.* 71.4%; 20/28) and agreement (absolute MNB <15% of 82.1%; 23/28 *vs.* 71.4%; 20/28), the same patterns for populations with lower degree of agreement (i.e., population defined based on limited and heterogeneous markers and infrequent subsets), were observed (**Table 3** and **Supplementary Table 6**).

**Table 3:** Reproducibility of manual analysis and automated database-guided analysis for the identification of all innate myeloid cell (IMC) and non-IMC populations (n=28) in EDTA-anticoagulated peripheral blood samples (n=6) stained with version 3 of the EuroFlow IMC tube.

Populations	Relative frequency from nucleated cells* (%)[median (min-max)]	E1 (1^st^ round) *vs*. E2 (1^st^ round)		E1 (1^st^ round) *vs*. E1 (2^nd^ round)		E1 (1^st^ round) *vs*. DB (1^st^ round)	
		R^2^	MNB (%)	R^2^	MNB (%)	R^2^	MNB (%)
**Debris/doublets**	NA	1.000	-1.8	0.998	-0.8	0.998	2.9
**Nucleated cells**	100	0.999	0.4	0.999	0.1	0.994	-0.8
**Unidentified cells**	33.9 (31.2 - 43.1)	0.999	0.4	1.000	0.2	0.994	-0.7
**Eosinophils**	3.0 (1.8 - 11.3)	1.000	-2.4	1.000	-0.07	0.996	-9.1
**Neutrophils**	51.8 (35.5 – 59.0)	1.000	0.7	1.000	0.1	0.998	-0.01
**Mature neutrophils**	51.8 (35.5 – 59.0)	1.000	0.7	1.000	0.1	0.998	-0.03
**Immature neutrophils**	0.06 (0.02 – 0.21)	0.998	-5.0	0.998	0.2	0.996	-0.8
** CD62L+ immature neutrophils**	0.01 (0.004 – 0.04)	0.993	-5.1	0.949	-9.1	0.985	-53.6
** CD62L- immature neutrophils**	0.04 (0.01 – 0.17)	0.999	-4.7	0.999	3.7	0.999	2.4
**Basophils**	0.7 (0.4 – 1.5)	0.995	2.1	0.997	-1.4	0.973	-5.3
**Monocytes**	9.2 (6.2 - 12.4)	0.998	0.8	0.999	0.2	0.979	-1.5
**cMo**	8.0 (5.5 – 9.9)	0.984	2.3	0.999	0.6	0.967	-2.8
** CD62L+ FcεRI- cMo**	5.4 (3.7 – 8.5)	0.742	-2.0	0.968	4.9	0.875	-0.4
** CD62L+ FcεRI+ cMo**	0.8 (0.08 – 1.9)	0.816	-31.6	0.882	-7.2	0.979	-17.2
** CD62L- FcεRI- cMo**	0.9 (0.4 – 1.0)	0.605	40.6	0.129	-28.6	0.108	-43.2
** CD62L- FcεRI+ cMo**	0.2 (0.02 – 0.5)	0.660	-47.6	0.871	-30.0	0.817	3.7
**iMo**	0.4 (0.2 – 1.0)	0.968	-30.2	0.983	1.2	0.962	13.5
**ncMo**	0.8 (0.5 – 2.1)	0.996	3.2	0.994	-4.7	0.978	-7.5
** CD36+ Slan- ncMo**	0.2 (0.10 – 0.4)	0.619	-0.9	0.710	15.6	0.749	-4.4
** CD36- Slan- ncMo**	0.2 (0.07 – 0.4)	0.930	46.1	0.968	-49.0	0.900	17.6
** CD36+ Slan+ ncMo**	0.01 (0.006 – 0.14)	0.951	56.3	0.916	6.0	0.988	-19.1
** CD36- Slan+ ncMo**	0.2 (0.1 – 1.3)	0.989	-25.9	0.990	4.3	0.994	-14.1
**CD1c+ myDCs**	0.3 (0.2 – 0.5)	0.780	-8.6	0.952	1.0	0.988	4.9
**CD1c+ CD14- myDCs**	0.2 (0.1 – 0.4)	0.975	3.3	0.998	-1.7	0.995	8.4
**CD1c+ CD14lo myDCs**	0.09 (0.05 – 0.2)	0.085	-31.8	0.671	-0.3	0.935	0.2
**CD141+ myDCs**	0.01 (0.004 - 0.03)	0.960	-11.7	0.995	2.4	0.999	2.3
**pDCs**	0.2 (0.03 – 0.5)	0.998	-8.1	0.997	3.6	1.000	2.0
**Axl^+^ DCs**	0.01 (0.004 – 0.03)	0.792	-1.3	0.614	-39.8	0.960	-23.75
**% of populations with R^2^ ≥ 0.9** **and p < 0.05 or -15% < MNB < +15%**		**71.4%** (20/28)	**71.4%** (20/28)	**78.6%** (22/28)	**82.1%** (23/28)	**85.7%** (24/28)	**82.1%** (23/28)

For determination of the comparability between analysis performed by two distinct experts (E1 vs. E2), at two distinct timepoints (2 months apart; 1^st^ round vs. 2^nd^ round) and between conventional manual and automated database-guided analysis, a linear regression was performed to evaluate the direction and strength of the relationship between the two conditions (high agreement defined by R^2^>0.9 and p<0.05). Additionally, a Bland-Altman analysis was done in order to determine the potential bias (high agreement defined as -15% > mean normalized bias (MNB) < +15%). *Median % of cells as identified by expert 1 (E1) (1^st^ round).

E1, experienced cytometrist 1; E2, novice cytometrist 2; DB, database-guided automated analysis; MNB, mean normalized bias (calculated as % of difference between conditions compared to the results of expert 1 – E1 - in the first round of analysis); cMo, classical monocytes; iMo, intermediate monocytes; ncMo, non-classical monocytes; DCs, dendritic cells; myDCs, myeloid DCs; pDCs, plasmacytoid DCs; NA, not applicable.

### Database construction and automated data analysis

Comparison of manual expert-based *vs.* database-guided automated gating showed a better degree of correlation (85.7%; 24/28) and agreement (82.1%; 23/28), compared to intra- and inter-operator manual analysis (**Table 3**), with an improved identification of some IMC populations defined based on the expression of heterogeneous markers (i.e., most of the ncMo subsets). Despite this, low correlation and/or degree of agreement was still observed for cMo subsets, defined based on the expression of CD62L and FcεRI, and IMC populations present at low frequency (<0.05%) such as Axl^+^ DC or CD62L^+^ immature neutrophils. Of note, database-guided automated gating and identification (AGI) performed at two different timepoints displayed a 100% correlation and degree of agreement for the 28 (IMC and non-IMC) populations tested, which clearly improves reproducibility compared to both intra- and inter-operator manual analysis.

### Age-related distribution of IMC populations in PB of HD

**Figure 9:**
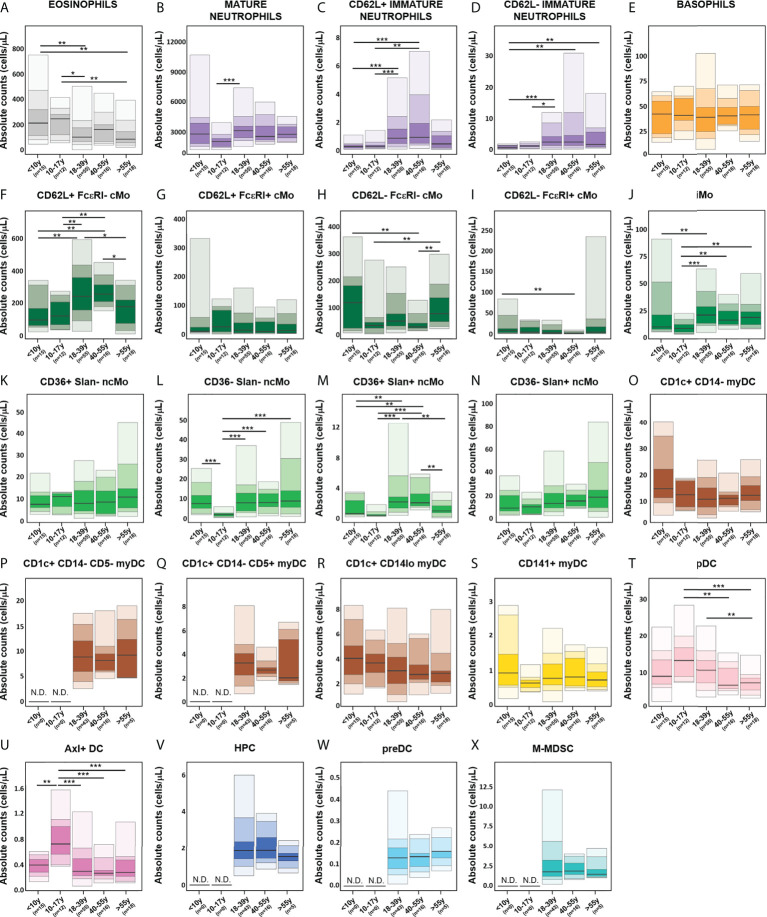
Age-related distribution of innate myeloid cell (IMC) populations identified using the EuroFlow IMC tube, based on the evaluation of blood samples from 116 healthy individuals. Reference ranges for all IMC populations identified with the innate myeloid (IMC) tube [granulocytic cells **(A–E)**, monocytic populations **(F–N)**, dendritic cell (DC) populations **(O–U)**, hematopoietic precursor cells (HPC) **(V)**, preDC **(W)** and monocytic myeloid-derived suppressor cells (M-MDSC) **(X)**] in different age categories. Minimum, percentiles 10, 25, 50 (median), 75 and 90, and maximum values are shown. Statistically significant differences were evaluated using a Kruskal-Wallis test and false discovery rate (FDR) of 5% to correct for multiple comparisons (* p-value < 0.05; ** p-value < 0.01; *** p-value < 0.001). cMo, classical monocytes; iMo, intermediate monocytes; ncMo, non-classical monocytes; DC, dendritic cells; myDC, myeloid dendritic cells; pDC, plasmacytoid dendritic cells; HPC, hematopoietic precursor cells; M-MDSC; monocytic myeloid-derived suppressor cells; N.D., not determined.

Overall, no significant age-related kinetics were observed for basophils, CD36^+^ Slan^-^ ncMo, CD141^+^ myDCs, HPC, M-MDSC and preDCs (**Figure 9**). In contrast, eosinophils and CD1c^+^ CD14^lo^ myDCs displayed decreased absolute counts in PB with age (**Figures 9A, R**), and CD36^-^ Slan^+^ ncMo tended to increase with ageing (**Figure 9N**). In turn, several IMC populations exhibited unique profiles around adolescence (10-17y), with CD62L^+^ FcεRI^+^ cMos, pDCs, Axl^+^ DCs and peaking in this age group (**Figures 9G, T, U**, respectively). Mature neutrophils, iMos and some populations of ncMos (CD36^-^ Slan^-^ and CD36^+^ Slan^+^ncMos) showed reduced numbers until the age of 10-17y with a rise again in young adults (18-39y) and stable numbers thereafter (**Figures 9B, J, L**), except for CD36^+^ Slan^+^ ncMos (**Figure 9M**) which decreased in the latter age group and CD62L^-^ FcεRI^-^ cMos (**Figure 9H**) which further increased in older adults (>55y). Conversely, CD62L^+^ FcεRI^-^ cMos peaked in young adults (18-39y), while they were reduced in older individuals (>55y) (**Figure 9F**), whereas CD1c^+^ CD14^-^ myDCs showed a decrease until 18-39y and remained stable thereafter (**Figure 9O**). Interestingly, both immature neutrophil populations (CD62L^-^ and CD62L^+^) displayed similar kinetics, peaking in middle aged adults (40-55y) and declining in older adults (>55y) (**Figures 9C, D**). Conversely, CD62L^-^ FcεRI^+^ cMos exhibited a decrease in absolute counts until the age of 40-55y, followed by a slight rise in older adults (**Figure 9I**).

When evaluating the potential impact of sex on the distribution of different IMC populations in PB, significantly lower mature neutrophil counts were observed in younger adults (18-39y) (**Supplementary Figure 9**) for men *vs.* women (p=0.04), whereas CD62L^-^ immature neutrophils (p=0.004) and CD62L^-^ cMo populations (CD62L^-^ FcεRI^-^ cMos, p=0.01; CD62L^-^ FcεRI^+^ cMos, p=0.008) were significantly increased in men *vs.* women.

## Discussion

Monitoring of IMC populations for diagnostic patient care has been historically hampered by the lack of standardized criteria for population identification and data analysis, coupled to the continuous developments in the field, with recent description of previously unknown (sub)populations of monocytes and DC (25, 26, 35, 40, 42, 44–46).

Here, we employed for the first time a stepwise unbiased approach for the development of two alternative antibody combinations for monitoring of up to 23 different IMC populations in normal PB, including recently described populations, such as Axl^+^ DCs and preDCs: an 11-color tube (with 13 antibodies), compatible with CE-IVD certified FCM instruments which identifies 19 IMC populations, and an extended 14-color variant (with 16 antibodies), allowing identification of 4 additional, less frequently reported, IMC populations. For fast translation to diagnostic laboratories, we evaluated the impact on both IMC population phenotypes and counts in PB, of different anticoagulants, immediate *vs.* delayed sample preparation and the usage of distinct types (conventional *vs.* spectral) of FCM instruments in single *vs.* multicenter settings. Finally, we developed a database-guided automated analysis approach for standardized data analysis and provided normal age- and sex-matched reference values as a basis for future immune-monitoring in patient care.

A backbone previously identified and validated by the EuroFlow and TiMaScan consortia for immune-monitoring of major granulocytic and monocytic (sub)populations (9, 37, 39, 67), was employed as a basis for panel design. This combination already allowed for identification of eosinophils, mature neutrophils, two populations of cMos (CD62L^+^ and CD62L^-^), iMo and four populations of ncMos (defined based on CD36 and Slan expression). Of note, previous reports suggested that CD9 instead of CD36 might also be used for ncMo subsetting within the Slan^+^ compartment (35). However, the expression of the two markers is redundant within Slan^+^ cells (35) and CD36 further allows for identification on an additional Slan^-^ ncMo population and at the same time, it is more specific for monocytes and DCs than CD9.

In a second step, markers classically employed for identification of pDCs (i.e., CD123, CD303 and CD304) (25, 27, 31, 41) and myDCs (i.e., CD11c and CD33) (38, 41, 42, 45, 68) were tested. CD303 and CD33 showed the best performance for clear discrimination of pDCs and myDCs, respectively, overcoming the need for an exclusion cocktail of lymphoid-associated markers. This is due to the fact that CD303 is highly specific for pDCs (69), and CD33 cross-contamination would result mainly from monocytes (70), which can be excluded based on counterstaining with the backbone markers. Other markers, e.g., CD11c are also expressed on B cells (71), and would require the inclusion of an exclusion B-cell marker. Although a splicing polymorphism has been reported for CD33, leading to loss of epitopes recognized by anti-CD33 antibodies (72), the usage of a bright fluorochrome (i.e., PE Cy7) in combination with other markers in the panel (e.g., FcεRI, CD14, CD16, CD1c, CD141, CD303) still allowed for accurate identification of myDCs, also in individuals displaying CD33^lo^ expression (data not shown).

Recent reports have highlighted the great heterogeneity of the myDC compartment (40, 42). For example, CD1c^+^ myDCs (or cDC2) are comprised of functionally distinct subsets that can be discriminated based on CD14 expression (CD14^lo^ inflammatory myDCs *vs.* CD14^-^ myDCs) (40). Likewise, Yin *et al.* (42) reported two populations of CD1c^+^ myDCs with distinct gene expression, cytokine production, migration potential, antigen presentation and T-cell polarization profiles, identified based on the expression of CD5^hi^ *vs.* CD5^lo^. Combining both markers allowed identification of three distinct populations of CD1c^+^ myDCs with the EuroFlow IMC tube: i) CD1c^+^ CD14^lo^ myDCs, ii) CD1c^+^ CD14^-^ CD5 myDCs ^-^ and iii) CD1c^+^ CD14^-^ CD5^+^ myDCs. Since both the CD14^lo^ and CD14^-^ CD5^-^ subsets of CD1c^+^ myDCs have been recently shown to display gene expression patterns closer to monocytes (40, 42), further transcriptomics, proteomics and/or functional comparative analyses are required to better understand the relationship among these subsets.

Classical gating strategies for pDCs identification have been associated with cross-contamination with the recently described Axl^+^ DCs (40). As these cells show myDC and pDC mixed transcriptomic and functional profiles, this could lead to potentially inaccurate data interpretation (40). Here we identified CD303^+^ Axl^+^ DC *vs.* pDCs and myDCs, in the absence of an anti-Axl antibody, based on a distinctive immunophenotypic profile (HLA-DR^+^ CD33^lo^ CD141^+^ CD303^lo^). This CD303^lo^ Axl^+^ DC population also showed unique functional features both at steady-state and in response to LPS. As described by Villani *et al* (40), the Axl^+^ DCs, here identified employing the above-mentioned combination, displayed higher CD86 and CD5 baseline expression *vs.* pDCs and produced IL6, IL8 and TNFα in response to TLR4 stimulation, with an intermediate degree of response between pDCs and CD1c^+^ myDCs, further supporting that Axl^+^ DCs can be identified based on the HLA-DR^+^ CD33^lo^ CD141^+^ CD303^lo^ phenotype. In addition to the pDC-like Axl^+^ DCs (CD11c^-/lo^, CD123^+^, Axl^+^), another Axl-expressing DC population has been reported in the literature (CD11c^+^ CD123^lo^ Axl^+^ DCs), which exhibits an immunophenotypic profile (CD11c^+^ CD14^-^ CD5^+^) (25, 40) similar to CD1c^+^ CD14^-^ CD5^+^myDCs. In line with this, both populations have also been reported to induce strong CD4^+^ T-cell proliferation (40, 42), suggesting that these two DC populations might be (at least partly) overlapping subsets. Further studies are required to confirm these observations.

While the nature of the myDCs precursor in PB is still a matter of debate (31, 40, 43), a population defined by a CD100^hi^ CD34^int^ phenotype, ability to proliferate and differentiate into CD1c^+^ myDCs and CD141^+^ myDCs has been reported (40). Remarkably, CD100 was not critically required for its identification since the HLA-DR^hi^ CD34^int^ phenotype showed a high discrimination power *vs.* other CD34^+^ cells. Interestingly, several recently described preDC populations, based on different antibody combinations, show significant overlapping features. For example, CD45RA^+^ CD33^+^ CD123^+^ HLA-DR^+^ preDCs described by See et al. (31) in fact correspond to Axl^+^ DC as proposed by Villani *et al.* (40) Altogether, these findings highlight the need for a standardized nomenclature of IMC populations for more direct comparison of data derived from different panels and studies.

Identification of immature neutrophil populations was accurately achieved using the backbone combination alone, which even allowed their further subsetting based on the pattern of expression of CD16 and CD62L. Interestingly, these populations displayed immunophenotypic features overlapping not only with immature neutrophil populations (promyelocytes, myelocytes and metamyelocytes) (63, 64), but also with polymorphonuclear myeloid-derived suppressor cells (PMN-MDSCs) (CD11b^+^, CD14^-^, CD15^+^, CD33^+^, CD66b^+^) (32), as previously reported by others (73–76). In fact, despite the guidelines for identification of PMN-MDSC *vs.* neutrophils require a standard density (e.g., Ficoll) gradient centrifugation step (32), previous groups have addressed the identification of PMN-MDSCs in whole blood (77). In line with our data, these groups also reported similar immunophenotypic profile to the one observed among immature neutrophils (CD3^-^, CD11b^+^, CD14, CD15^+^, CD16^-^, CD19^-^, CD20^-^, CD33^+^, CD45^+^, CD56^-^, CD45+, HLA-DR^-^) (**Figure 4**). Furthermore, as previously reported for PMN-MDSCs (78), an increased frequency of immature neutrophils was observed in CB *vs.* adult PB, further supporting the notion that these might be (at least in part) overlapping IMC populations. Further functional, immunophenotypic, biochemical and molecular studies (e.g., inhibition of T-cell proliferation, reactive oxygen species production or expression of Arginase 1, Lox-1 or VEGFR1) (32, 79, 80) in e.g. PB from cancer patients are required to determine the degree of overlap between these populations and which additional markers would potentially be required to differentiate them.

Discrimination of M-MDSCs from cMos frequently depends solely on the pattern of expression of HLA-DR, which ultimately requires FMO or internal controls to set the gates for their arbitrary identification (32). While several studies have reported markers with the potential to improve the discrimination from cMos (e.g., CD64, CD86, CD124, CD163, S100A9) (32, 81), no comprehensive evaluation of the expression of high numbers (n>30) of proteins in cMos *vs.* M-MDSCs has been previously performed. In line with earlier reports (32, 81, 82), a trend for lower expression of CD32, CD64, CD86 and CD163 and increased expression of CD124 and S100A9 was observed in M-MDSCs *vs.* cMos. Despite this, only CD16, CD123 and CD192 showed overall statistically significant different expression in M-MDSCs *vs.* cMos. This might be due to the fact that normal CB and healthy adult PB samples were tested in our study, whereas other reports evaluated these markers in cancer, infection and/or inflammatory conditions (81, 82), that can potentially lead to more pronounced distinct phenotypes. Multivariate analysis further revealed that only CD192 was of additional value for discrimination of the two populations and therefore, only this marker was included in the extended version of the EuroFlow IMC tube. Interestingly, when CD192 was used, a significantly higher frequency of M-MDSC was observed in CB *vs.* adult PB, a pattern previously reported for PMN-MDSCs but not M-MDSCs (78), suggesting that the more restricted CD14^+^, HLA-DR^-/lo^, CD192^-/lo^ phenotype could potentially more accurately identify CD14^+^ HLA-DR^-/lo^ M-MDSCs. Further T-cell proliferation inhibition assays are required to confirm this hypothesis. Based on all the above, we can conclude that the number of markers required to identify all distinct target populations of IMC was optimized in the EuroFlow IMC combinations.

For increased flexibility, two versions of the EuroFlow IMC tube were designed. A more limited, smaller 11-color antibody combination (13 antibodies), aimed for the clinical setting, in which available IVD-certified instruments frequently have the ability to detect fewer parameters, and an extended 14-color tube (16 antibodies), that further allows identification of less frequent and/or more recently discovered IMC populations (e.g., M-MDSCs and preDCs), mostly aimed at the discovery/research settings, in which instruments allowing simultaneous detection of >12 colors are more commonly available.

In line with previous reports (66, 83), both antibody combinations can be used in EDTA *vs.* sodium heparin anticoagulated samples, although slightly lower counts of CD1c^+^ CD14^lo^ myDCs might be detected in heparin samples. Similarly, no significant impact on the overall staining patterns and individual marker resolution was observed for samples stored at RT for up to 24h prior to staining, except for lower CD16 and Slan levels, according to previously reported findings for CD16 (66). However, an increasing time lapse between sample collection and sample processing had a significant impact on the absolute counts of specific IMC populations, already at >12 hours and particularly at ≥ 24h, when >60% of all IMC populations evaluated exhibited some degree of altered (>10% variation *vs.* 0h) cell counts, in line with previous studies (66, 77). However, it should be noted that delayed sample preparation mainly affected infrequent populations (e.g., Axl^+^ DCs, CD1c^+^ CD14^dim^ myDCs, CD141^+^ myDCs), leading to an overestimation of their counts, which might be due to the lower viability of more frequent populations, as supported by an increased percentage of cell debris, particularly at 24h. Conversely, underestimation of populations of (particularly CD36^-^) ncMos was observed after 12h, probably because ncMos have been reported to be more prone to spontaneous apoptosis (84). Interestingly, CD62L^-^ cMos were more sensitive to delayed processing than CD62L^+^ cMos. Downregulation of CD62L by mechanisms such as cleavage from the cell surface membrane has been shown in apoptotic mature neutrophils (85). A similar process might occur in monocytes. Of note, our time course experiments were performed at RT, aiming at mimicking transportation of the samples between centers. However, the performance of the EuroFlow IMC tube could be improved by storage/transportation of samples at 4°C in sodium heparin-anticoagulated tubes, as recommended by Diks *et al.* (66) who reported good stability of major myeloid populations up to 24h under these conditions. A frequently employed alternative approach for the study of samples that cannot be evaluated within a short period upon collection is freezing. However, while the overall staining resolution of samples with the EuroFlow IMM combination was not significantly affected by the freezing process, and still allowed for identification of all IMC populations present in the sample, a clear impact on the relative frequency of populations was observed. Overall, this suggests, that despite the combination can be employed for characterization of frozen PBMCs, in the context of comparison of samples processed with the same method, the interpretation and reporting of the results on relative frequency of populations should consider the bias *vs.* freshly obtained samples induced by freezing procedure.

Further evaluation of the EuroFlow IMC tube showed a very good reproducibility both in single center, multi-instrument, and multi-center settings. Of note, the highly comparable results obtained in conventional *vs.* spectral instruments support the possibility of employing the EuroFlow IMC tube as a basis for expansion with additional application-dependent characterization markers, when high-end (>20 colors) instruments are used in a research setting. Noteworthy, as the frequency of some of the IMC population can be as low as 0.1 cells/μL in healthy donors, to reliably and reproducibility identify and quantify these populations also in situations in which a significantly decreased frequency is observed, staining of 10^7^ cells is recommended. While the EuroFlow IMC combination can be employed for processing of lower numbers of cells, in case of limited sample availability, the limits of detection (LOD) and quantitation (LOQ) (≥30 and ≥50 events to define a cell population, respectively) should be taken into account for data analysis and reporting.

A high correlation between automated *vs.* expert-based manual analysis was observed for population identification and quantification, in line with previous reports (56, 57, 86). The higher reproducibility observed for repeated database-guided AGI procedures *vs.* expert-based manual analysis, together with the faster (approximately 5min *vs.* 20min for analysis of one sample, respectively) and less labor-intensive features of AGI, further support the potential of database-guided automated analysis to reduce operator-related variability and allow for more efficient and reproducible data analysis. These features become particularly relevant in the diagnostic clinical setting and in cases where a high number of parameters and/or IMC populations are investigated (57, 86, 87). Interestingly, less than optimal performance observed for database-guided automated analysis was restricted to the analysis of minimally represented IMC populations (<0.05% of all leukocytes) close to the limit of quantification (LOQ) of the tube, and populations defined by a limited number of gating markers with heterogeneous expression patterns (e.g., ncMo or cMo subsets). Improvement of the performance of the database-guided analytical procedures might be potentially achieved by staining and acquisition of higher numbers of cells (e.g., 10 million) and fine-tuning of Wanderlust trajectory-based automated gating on heterogenous markers (50, 86).

The frequency of IMC populations has been previously shown to be modulated throughout life (37). Therefore, knowledge of the normal age-related distribution of the populations is crucial for clinical translation of the data. Overall, three major patterns were observed for the absolute counts of PB IMC populations in relation with age: i) stable cell counts, ii) modulation during adolescence and iii) changes in older (>55y) adults. Since the distribution of several immune cell populations has been reported to occur within the first 2 years of life most prominently (37, 86), it is possible that earlier kinetic changes in populations have been missed, as our cohort only includes children >8 years. Further inclusion of samples from younger infants would allow for applicability of the reference values in the pediatric settings. Overall, several populations displayed clear kinetics around adolescence (10-17y) (e.g., pDCs, Axl^+^ DCs and CD36^-^/Slan^-^ ncMos), most likely associated with the physiological changes observed in puberty (e.g., hormonal variations and increased tissue remodeling). In turn, other IMC populations showed modulation in older adults (e.g. eosinophils, immature neutrophils, CD62L^+^ FcεRI^-^ cMos and CD36^+^ Slan^+^ ncMos), potentially as a result of a skewing of hematopoiesis towards myeloid *vs.* lymphoid lineages, decrease in the function of neutrophil, monocytes and DCs and possibly also low-grade inflammation also known as “inflamm-aging” (88, 89).

In contrast to age, limited sex-related differences were observed, except for the more mature neutrophils (more frequent in women), immature neutrophils and CD62L^-^ cMos (more frequent in men), similarly to what has been previously reported for neutrophils and lymphocytes (90, 91).

In summary, we developed two standardized, and highly reproducible versions of the EuroFlow IMC tube, which are suitable for clinical and research/discovery studies, even in multi-instrument and multi-center settings, allowing for robust and accurate identification and quantitation of 19 to 23 IMC populations in blood. By addressing distinct (i.e., pre-analytical, analytical and post-analytical) variables that might impact the reproducibility of laboratory testing, and providing normal age- and sex-related reference ranges, our study sets the basis for standardized immune-monitoring of IMC in distinct disease and treatment conditions, in the context of clinical trials and/or patient care such as in inflammatory diseases, various forms of tissue damage as well as for monitoring immune responses to infectious diseases, vaccination or immunotherapy.

## Data availability statement

The raw data supporting the conclusions of this article will be made available by the authors, without undue reservation.

## Ethics statement

The studies involving human participants were reviewed and approved by Medical Research Ethics Committees United (MEC-U, NL60807.100.17-R17.039), Medisch Ethische Toetsingscommissie (METC) – LUMC, Comité de Ética de la Investigación con medicamentos del Área de Salud de Salamanca. Written informed consent to participate in this study was provided by the participants’ legal guardian/next of kin.

## Author contributions

CT, AO, and JD designed the study. KP, SB-V, DD, WB, IL, BN, PD, SK, AL, and AJ performed the flow cytometry experiments. KP and IL performed the *in vitro* activation assays. GF, provided the cord blood samples. DD, AH-D, JA, JVG, A-MB, QH, AO, and CT were involved in the multicentric validation. KP, CT, IL, BN, PD, AJ, A-MD, MB, BM, PV, GB and RG contributed to the collection, processing and/or analysis of samples of healthy donors for calculation of the reference values. AS-G, FB, A-MD, MB, BM, and RG performed the technical evaluation of the tube. AH-D, DD, and CT constructed and validated the database for automated analysis. CT, KP, and IK made the figures and performed the statistical analyses. KP, CT, and JD wrote the manuscript. All authors contributed to manuscript revision and approved the submitted version.

## Funding

The presented work was funded by the European Research Council under the European Union’s Horizon 2020 Research and Innovation Programme with an ERC Advanced Grant (ERC-2015-AdG 695655, TiMaScan) and the IMI2 PERISCOPE project, financed by the Innovative Medicines Initiative 2 Joint Undertaking (grant number 115910). This joint undertaking receives support from the European Union’s Horizon 2020 Research and Innovation Programme, the European Federation of Pharmaceutical Industries and Associations (EFPIA), and The Bill and Melinda Gates Foundation (BMGF). This manuscript only reflects the author’s views. The Joint Undertaking is not responsible for any use that may be made of the information this manuscript contains. The coordination and innovation processes of this study were supported by the EuroFlow Consortium. The EuroFlow Consortium received support from the FP6-2004-LIFESCIHEALTH-5 program of the European Commission (grant LSHB-CT-2006-018708) as Specific Targeted Research Project (STREP).

## Acknowledgments

We would like to acknowledge and thank the LUMC Vrijwillige Donoren Service – Direct Gebruik (LuVDS-DG; B18.031) and donors for supplying the peripheral blood samples (request code L18.001) and the Flow cytometry Core Facility (FCF) of the Leiden University Medical Center (LUMC), The Netherlands (https://www.lumc.nl/research/facilities/fcf), coordinated by M. Hameetman, run by the FCF operators, D.M. Lowie, S. van de Pas, G.IJ. Reyneveld, and former FCF coordinator dr. K Schepers and former operators J.P. Jansen, E.F.E de Haas, G.M. De Roo, R.J. Mclaughlin and S.A.J. Veld (Directors: Prof. F.J.T. Staal and Prof. J.J.M. van Dongen) for their technical support.

## Conflict of interest

JD and AO report to be chairmen of the EuroFlow scientific foundation, which receives royalties from licensed patents, which are collectively owned by the participants of the EuroFlow Foundation. These royalties are exclusively used for continuation of the EuroFlow collaboration and sustainability of the EuroFlow consortium. JD and AO report an Educational Services Agreement from BD Biosciences (San José, CA) and a Scientific Advisor Agreement with Cytognos; all related fees and honoraria are for the involved university departments at Leiden University Medical Center and University of Salamanca. AH-D is an employee of Cytognos (Salamanca, Spain). Lastly, JD, CT, AO, JA, WB, KP, MB, AD, DD, and AH-D, are listed as (co)inventors on the patent “Means and methods for multiparameter cytometry-based leukocyte subsetting” (NL2844751, filing date 5 November 2019), owned by the EuroFlow scientific consortium, which describes the flow cytometry panels developed in this study.

The remaining authors declare that the research was conducted in the absence of any commercial or financial relationships that could be construed as a potential conflict of interest.

## Publisher’s note

All claims expressed in this article are solely those of the authors and do not necessarily represent those of their affiliated organizations, or those of the publisher, the editors and the reviewers. Any product that may be evaluated in this article, or claim that may be made by its manufacturer, is not guaranteed or endorsed by the publisher.
