# Supplementary material for: Development of a standardized and validated flow cytometry approach for monitoring of innate myeloid immune cells in human blood
Source: Front Immunol. 2022 Sep 14;13:935879. doi: 10.3389/fimmu.2022.935879 (PMC9519388; doi:10.3389/fimmu.2022.935879)
Supplement: Supplementary file 1 [file DataSheet_1.zip › van der Pan et al_EuroFlow immune-monitoring innate myeloid-cell tube_Supplementary Material, Methods and Results.docx]

Supplementary Material

**Development of a standardized and validated flow cytometry approach for monitoring of innate myeloid immune cells in human blood**

Kyra van der Pan,^1^ Sandra de Bruin-Versteeg,^1^ Daniela Damasceno,^2^ Alejandro Hernández-Delgado,^2^ Alita J van der Sluijs-Gelling,^1^ Wouter BL van den Bossche,^1,3^ Inge F de Laat,^1^ Paula Díez,^1^ Brigitta AE Naber,^1^ Annieck M Diks,^1^ Magdalena A Berkowska,^1^ Bas de Mooij,^1^ Rick J Groenland,^1^ Fenna J de Bie,^1^ Indu Khatri,^1^ Sara Kassem,^1^ Anniek L de Jager,^1^ Alesha Louis,^1^ Julia Almeida,^2^ Jacqueline AM van Gaans-van den Brink,^4^ Alex-Mikael Barkoff,^5^ Qiushui He,^5^ Gerben Ferwerda,^6^ Pauline Versteegen,^4^ Guy AM Berbers,^4^ Alberto Orfao,^2^ Jacques JM van Dongen,^1,2*^ Cristina Teodosio,^1,2^ on behalf of the TiMaScan, IMI2 PERISCOPE and EuroFlow Consortia.

**Institutional affiliations:**

^1^ Dept. of Immunology, Leiden University Medical Center, Leiden, The Netherlands

^2^ Translational and Clinical Research Program, Cancer Research Center (IBMCC; University of Salamanca - CSIC); Cytometry Service, NUCLEUS; Department of Medicine, University of Salamanca (Universidad de Salamanca, ROR https://ror.org/02f40zc51) and Institute of Biomedical Research of Salamanca (IBSAL), Salamanca, Spain

^3^ Dept. of Immunology and Department of Neurosurgery, Brain Tumor center, Erasmus Medical Center, University Medical Center Rotterdam, Rotterdam, The Netherlands

^4^ Centre for Infectious Disease Control, National Institute for Public Health and the Environment (RIVM), Bilthoven, The Netherlands

^5^ Institute of Biomedicine, Research Center for Infections and Immunity, University of Turku (UTU), Turku, Finland

^6^ Section of Paediatric Infectious Diseases, Laboratory of Medical Immunology, Radboud Institute for Molecular Life Sciences, Nijmegen, The Netherlands

***Correspondence:** Prof. J.J.M. van Dongen, MD, PhD

Leiden University Medical Center (LUMC)

J.J.M.van_Dongen@lumc.nl

**Keywords:** immune-monitoring, flow cytometry, innate myeloid cells, age-related reference values, standardization

**Running title (5 words):** EuroFlow immune-monitoring innate myeloid-cell tube

**SUPPLEMENTARY MATERIAL AND METHODS**

**Immunophenotypic studies on whole blood samples.** For every flow cytometric assay performed, EuroFlow standard operating procedures (SOPs) for sample preparation and staining were used.(1) Briefly, peripheral blood (PB) was incubated with ammonium chloride solution (NH_4_Cl) for 15 min to lyse red blood cells and cells were washed twice with phosphate buffered saline (PBS) containing 0.5% bovine serum albumin (BSA), 0.1% sodium azide (NaAz) and 2 mM EDTA (pH=7.4). Nucleated cells were counted using a Sysmex XP-300 (Sysmex Europe GmbH, Norderstedt, Germany) automated hematological analyzer and 10^7^ leukocytes were incubated in the dark for 30 min, rolling, at room temperature (RT) with the antibodies directed against surface membrane (Sm) (Table 1). For staining of Sm markers only, 2 ml FACS lysing solution (BD Biosciences, San Jose, CA) were then added and another incubation for 10 min at RT was performed. Subsequently, cells were washed once and resuspended in 500 μL of PBS.

For Sm plus intracellular (Cy) stainings, cells stained for Sm antibodies as described above, were subsequently incubated (15min, RT) with 100 μL of reagent A of the Fix&Perm™ Cell Permeabilization Kit (Thermo Fisher Scientific, Waltham, MA). Afterward, cells were washed and resuspended in 100 μL of reagent B of the Fix&Perm™ Cell Permeabilization Kit and incubated for another 15 min in the presence of antibodies directed against Cy markers. Stained cells were washed once in PBS and resuspended in 500 μL of PBS.

**Isolation, freezing and staining peripheral blood mononuclear cells (PBMCs).** Isolation of PBMCs was performed by density gradient centrifugation using Ficoll-Paque Plus (GE Healthcare Bio-Sciences AB, Uppsala, Sweden), according to the manufacturer’s instructions. Upon isolation, cells were washed twice with PBS and resuspended in RPMI 1640 medium (Sigma-Aldrich, Zwijndrecht, The Netherlands). Part of the sample was used for immediate staining, while the rest was frozen for posterior analysis. Freezing was performed by mixing one volume of the cell suspension with one volume of a freezing mix consisting of 20% Dimethyl Sulfoxide (DMSO) (Sigma-Aldrich) diluted in RPMI 1640 medium (Sigma-Aldrich), resulting in a final DMSO concentration of 10%. The sample was then frozen using a temperature gradient and stored in liquid nitrogen for one week before thawing and further processing.

For sample staining, 10^7^ PBMCs were incubated in the dark for 30 min at RT with the antibodies from version 4 of the EuroFlow IMC tube (Table I) and washed with PBS. Staining with Zombie NIR Fixable Viability Staining (Biolegend, San Diego, CA, USA) was then performed according to the manufacturer’s protocol for 30 min at RT, followed by incubation with 2 ml FACS lysing solution (BD Biosciences) for 10 min at RT. Subsequently, cells were washed once and resuspended in 500 μL of PBS.

**Database construction and inclusion criteria for .FCS files.** Selection of flow cytometry standard (.FCS) files to be employed for database construction was based on i) percentage of cell debris/doublets <25%; ii) stable acquisition during time; iii) ≥3x10^6^ leukocytes acquired; iv) FSC (forward scatter) and SSC (side scatter) detectors set so that lymphocytes complied with a reference target channel (arbitrary units) of 55,000 (range: 50,000-60,000) and 13,000 (range: 11,000-15,000), respectively; vi) use of the appropriate fluorochrome-specific compensation matrix; and vii) optimal staining, detected based on internal negative and positive controls. Samples with deviation from more than one condition and/or critical deviation for one criterion were excluded from the database.

With those 18 samples that fulfilled all the selection criteria, a database was built using a strategy previously described.(2)

Database-guided automated population gating and identification, implemented in Infinicyt^TM^ (Cytognos S.L., Salamanca, Spain) software, was performed based on a set of steps. Firstly, automated gating was established based on unsupervised clustering algorithms (minimum 10 events required per cluster at a maximum distance/dispersion within the cluster of 0.9), as previously described in detail.(2, 3) In a second step, a quantitative comparison of the immunophenotypic profiles of each cluster was performed with each cell population previously included in the reference database, using principal component and canonical clustering multiparameter analyses. Lastly, the clusters were classified as phenotypically identical or not to a cell population. Accordingly, clusters fully matching (i.e., falling within 2.5*standard deviations) of the immunophenotypic profile of said cell population in the reference database were classified as that cell population. Conversely, clusters showing phenotypic differences *vs.* the populations present in the reference database were signalled to be checked by an expert cytometrist.

**Statistical analysis.** Median, range, mean and standard deviation (SD) values and both the 25^th^ and 75^th^, and the 10^th^ and 90^th^ percentiles, were calculated for continuous variables; categorical variables frequencies were reported. The degree of association between two continuous variables was evaluated using linear regression analysis, and the degree of agreement between them was assessed by Bland-Altman plots. Statistical significance (p value <0.05) was determined by the non-parametric Kruskal-Wallis and Mann-Whitney U tests (for continuous variables in case of independent samples), or the Wilcoxon's nonparametric test (for paired samples) based on a false discovery rate of <5% to correct for multiple comparisons (Benjamini and Hochberg method). For all statistical analyses the GraphPad Prism 8.0 software (GraphPad, San Diego, CA) was used.

For multivariate visualization of the overall expression profile for activation- and maturation-associated markers which showed statistically significantly different expression patterns in resting steady-state vs. LPS stimulated cells, data on each population was normalized as described elsewhere.(4) Principal component analysis (PCA), canonical multivariate analysis (CA), Wanderlust trajectory analyses and t-distributed stochastic neighbour embedding (tSNE) analysis were performed using Infinicyt^TM^ (Cytognos S.L.). Unsupervised hierarchical clustering analysis was performed using the average fold change between stimulated and unstimulated conditions per marker, employing the Euclidean distance measure and average linkage clustering method in MultiExperiment Viewer (MeV) v.4.9.0 software. (5)

**REFERENCES**

1. Kalina T, Flores-Montero J, van der Velden VH, Martin-Ayuso M, Bottcher S, Ritgen M, et al. EuroFlow standardization of flow cytometer instrument settings and immunophenotyping protocols. Leukemia. 2012;26(9):1986-2010.

2. Flores-Montero J, Grigore G, Fluxa R, Hernandez J, Fernandez P, Almeida J, et al. EuroFlow Lymphoid Screening Tube (LST) data base for automated identification of blood lymphocyte subsets. J Immunol Methods. 2019;475:112662.

3. Fluxá Rodriguez R, Orfao De Matos Correia E Valle A, Hernández Herrero J.B., Method of digital information classification. US10133962B2 (Patent) 2017

4. van den Heuvel D, Jansen MAE, Nasserinejad K, Dik WA, van Lochem EG, Bakker-Jonges LE, et al. Effects of nongenetic factors on immune cell dynamics in early childhood: The Generation R Study. The Journal of allergy and clinical immunology. 2017;139(6):1923-34 e17.

5. Saeed AI, Sharov V, White J, Li J, Liang W, Bhagabati N, et al. TM4: a free, open-source system for microarray data management and analysis. Biotechniques. 2003;34(2):374-8.

**SUPPLEMENTARY TABLE 1.** Monoclonal antibodies employed for the design and construction of the EuroFlow Innate Myeloid Cell (IMC) tube.

| **Marker** | **Fluorochrome** | **Clone** | **Company** | **Antibody Identifier** | **Marker** | **Fluorochrome** | **Clone** | **Company** | **Antibody Identifier** |
| --- | --- | --- | --- | --- | --- | --- | --- | --- | --- |
| **Axl** | PE | 108724 | R&D Systems | Cat# FAB1541P, RRID:AB_2044610 | **CD63** | PE CF594 | H5C6 | BD Biosciences | Cat# 565403, RRID:AB_2739220 |
| **CD1c** | BB515 | F10/21A3 | BD Biosciences | Cat# 565054, RRID:AB_2716870 |  | PE | MEM-259 | Antibodies Online | Cat# ABIN94216, RRID:AB_10762536 |
|  | PerCP Cy5.5 | F10/21A3 | BD Biosciences | Cat# 565423, RRID:AB_2744320 | **CD64** | PerCP Cy5.5 | 10.1 | BD Biosciences | Cat# 561194, RRID:AB_10563216 |
|  | FITC | L161 | BD Biosciences | Cat# 331518, RRID:AB_2073403 | **CD68** | FITC | Ki-m7 | ThermoFisher | Cat# MA1-82715, RRID:AB_929275 |
| **CD5** | BV510 | UCHT2 | BD Biosciences | Cat# 340658, RRID:AB_400528 | **CD69** | BV650 | FN50 | BD Biosciences | Cat# 563835, RRID:AB_2738442 |
|  | PE Cy7 | L17F12 | BD Biosciences | Cat# 348810, RRID:AB_2848145 | **CD83** | PE CF594 | HB15e | BD Biosciences | Cat# 562631, RRID:AB_2737688 |
| **CD11b** | AF700 | IRCF 44 (44) | BD Biosciences | Cat# 557918, RRID:AB_396939 | **CD86** | BV650 | 2331 (FUN-1) | BD Biosciences | Cat# 563411, RRID:AB_2744456 |
|  | PE CF594 | IRCF 44 (44) | BD Biosciences | Cat# 562399, RRID:AB_2737613 | **CD100** | FITC | REA316 | Miltenyi Biotech | Cat# 130-104-672, RRID:AB_2654319 |
|  | PE | D12 | BD Biosciences | Cat# 333142, RRID:AB_2868643 | **CD107a** | APC R700 | H4A3 | BD Biosciences | Cat# 565184, RRID:AB_2739098 |
| **CD11c** | BV650 | b-ly6 | BD Biosciences | Cat# 563403, RRID:AB_2732048 | **CD123** | FITC | AC145 | Miltenyi Biotech | Cat# 130-090-897, RRID:AB_244210 |
|  | PE | b-ly6 | BD Biosciences | Cat# 560999, RRID:AB_2033937 |  | PE | 7G3 | BD Biosciences | Cat# 561058, RRID:AB_10584318 |
| **CD13** | PE | WM15 | BD Biosciences | Cat# 555394, RRID:AB_395795 | **CD124** | PE | G077F6 | Biolegend | Cat# 355004, RRID:AB_11219385 |
| **CD14** | APC-H7 | MφP9 | BD Biosciences | Cat# 641394, RRID:AB_1645725 | **CD141** | BV421 | 1A4 | BD Biosciences | Cat# 565321, RRID:AB_2739180 |
|  | PE CF594 | MφP9 | BD Biosciences | Cat# 562335, RRID:AB_11153663 |  | BV510 | 1A4 | BD Biosciences | Cat# 563298, RRID:AB_2728103 |
|  | PerCP Cy5.5 | M5E2 | Biolegend | Cat# 301823, RRID:AB_893253 | **CD163** | PE | GHI/61 | BD Biosciences | Cat# 556018, RRID:AB_396296 |
| **CD15** | BV510 | W6D3 | BD Biosciences | Cat# 563141, RRID:AB_2738025 | **CD192** | BV421 | K036C2 | Biolegend | Cat# 357210, RRID:AB_2563463 |
| **CD16** | AF700 | 3G8 | BD Biosciences | Cat# 560713, RRID:AB_1727430 |  | BV605 | K036C2 | Biolegend | Cat# 357214, RRID:AB_2563876 |
|  | BV786 | 3G8 | BD Biosciences | Cat# 563689, RRID:AB_2744299 | **CD197** | PE CF594 | 150503 | BD Biosciences | Cat# 562381, RRID:AB_11153301 |
|  | PE Cy7 | 3G8 | BD Biosciences | Cat# 557744, RRID:AB_396850 | **CD206** | BV650 | 19.2 | BD Biosciences | Cat# 740598, RRID:AB_2740298 |
| **CD19** | BB515 | HIB19 | BD Biosciences | Cat# 564456, RRID:AB_2744309 | **CD244** | PE | REA112 | Miltenyi Biotech | Cat# 130-099-071, RRID:AB_2656608 |
|  | BV605 | HIB19 | Biolegend | Cat# 302244, RRID:AB_2562015 | **CD300e** | APC | UP-H2 | Immunostep | Cat# IREM2A-100T, RRID:AB_11140615 |
|  | PE Cy7 | J3-119 | Beckman Coulter | Cat# IM3628U, RRID:AB_10638575 |  | APC C750 | UP-H2 | Immunostep | Cat# IREM2AC750, RRID:AB_2848148 |
| **CD25** | BV650 | M-A251 | BD Biosciences | Cat# 563718, RRID:AB_2744337 | **CD303** | APC | AC144 | Miltenyi Biotech | Cat# 130-090-905, RRID:AB_244165 |
| **CD32** | BV421 | FLI8.26 | BD Biosciences | Cat# 564838, RRID:AB_2738976 |  | PE | AC144 | Miltenyi Biotech | Cat# 130-090-511, RRID:AB_244168 |
| **CD33** | BV650 | WM53 | Biolegend | Cat# 303430, RRID:AB_2650934 | **CD304** | APC | 12C2 | Biolegend | Cat# 354506, RRID:AB_11219600 |
|  | BV785 | WM53 | Biolegend | Cat# 303428, RRID:AB_2650888 | **CD312** | PE | REA302 | Miltenyi Biotec | Cat# 130-104-583, RRID:AB_2657346 |
|  | PE Cy7 | P67.6 | BD Biosciences | Cat# 333952, RRID:AB_2713932 | **FcεRI** | FITC | AER-37 | Thermo Fisher | Cat# 11-5899-41, RRID:AB_10732343 |
|  | PE | P67.6 | BD Biosciences | Cat# 345799, RRID:AB_2868823 |  | PE | AER-37 | Thermo Fisher | Cat# 12-5899-42, RRID:AB_10804885 |
|  | PerCP Cy5.5 | P67.6 | BD Biosciences | Cat# 333146, RRID:AB_286864 | **HLA-DR** | BV711 | G46-6 | BD Biosciences | Cat# 564041, RRID:AB_2738559 |
| **CD34** | PE CF594 | 581 | BD Biosciences | Cat# 562383, RRID:AB_11154586 | **IL1β** | PE | AS10 | BD Biosciences | Cat# 340516, RRID:AB_400439 |
| **CD35** | PE | E11 | BD Biosciences | Cat# 559872, RRID:AB_397352 | **IL-6** | PE | MQ2-6A3 | BD Biosciences | Cat# 559331, RRID:AB_397228 |
| **CD36** | BV605 | CB38 | BD Biosciences | Cat# 563518, RRID:AB_2738250 | **IL-8** | PE Cy7 | E8N1 | Biolegend | Cat# 511416, RRID:AB_2565291 |
|  | PerCP Cy5.5 | CLB-IVC7 | Immunostep | Cat# 36PP5.52, RRID:AB_2848146 | **IL-10** | PE Cy7 | JES3-9D7 | Biolegend | Cat# 501420, RRID:AB_2125385 |
| **CD45** | AF700 | HI30 | BD Biosciences | Cat# 560566, RRID:AB_1645452 | **IL-12** | BV421 | C8.6 | BD Biosciences | Cat# 565023, RRID:AB_2739045 |
|  | OC515 | GA90 | Cytognos | Cat# CYT-45OC, RRID:AB_2848147 | **IL-13** | PerCP Cy5.5 | JES10-5A2 | Biolegend | Cat# 501911, RRID:AB_2124284 |
|  | PacO | HI30 | ThermoFisher | Cat# MHCD4530, RRID:AB_10376143 | **MPO** | PE | MPO-7 | Agilent DAKO | Cat# R720901, RRID:AB_579575 |
| **CD45RA** | PE | HI100 | Biolegend | Cat# 304108, RRID:AB_314412 | **Slan** | PE | M-DC8 | Miltenyi biotech | Cat# 130-093-029, RRID:AB_871582 |
| **CD62L** | BV605 | DREG-56 | Biolegend | Cat# 304834, RRID:AB_2562130 | **S100A9** | FITC | REA859 | Miltenyi Biotech | Cat# 130-114-706, RRID:AB_2726767 |
|  | BV650 | DREG-56 | Biolegend | Cat# 304832, RRID:AB_2563821 | **TGFβ** | BV421 | TW-9E7 | BD Biosciences | Cat# 562962, RRID:AB_2737919 |
|  |  |  |  |  | **TNFα** | PerCP Cy5.5 | Mab11 | Biolegend | Cat# 502926, RRID:AB_2204081 |

**Abbreviations:** RRID, Research Resource Identifier; Cat#, catalog number; PE, Phycoerythrin; BB, Brilliant Blue; PerCP Cy5.5, Peridinin-chlorophyll-protein-cyanin 5.5; BV, Brilliant Violet; Cy7, Cyanin7; AF700, Alexa Fluor 700; CF, Cyanin-based Fluorescent dye; APC, allophycocyanin; H7, hilite7; OC, Orange Cytognos; FITC, Fluorescein isothiocyanate; PacO, Pacific Orange.

**SUPPLEMENTARY TABLE 2.** Antibody combinations used for functional validation Axl^+^ dendritic cells analysed employing the combined antibody backbone selected for their immunophenotypic identification in the EuroFlow Innate Myeloid Cell (IMC) tube.

|  | BV421 | OC515/ BV510 | BV605 | BV650 | BV711 | BV786 | FITC/ BB515 | PerCP Cy5.5 | PE | PE CF594 | PE Cy7 | APC | AF700 | APC H7 | Samples evaluated  (n=) |
| --- | --- | --- | --- | --- | --- | --- | --- | --- | --- | --- | --- | --- | --- | --- | --- |
| Axl^+^ DC validation- phenotype | CD141 | CD45 OC515 | CD62L | CD69 | HLA-DR | CD16 | CD1c  BB515 | - | FcεRI | CD63 | CD33 | CD300e  +  CD303 | CD107a | CD14 | 4 |
|  | CD141 | CD45 OC515 | CD62L | CD86 | HLA-DR | CD16 | CD1c  BB515 | - | FcεRI | CD83 | CD33 | CD300e  +  CD303 | CD11b | CD14 |  |
| Axl^+^ DC validation- cytokine production | cyTGFβ | CD141  BV510 | CD62L | CD33 | HLA-DR | CD16 | FcεRI  FITC | cyTNFα | cyIL-1β | - | cyIL-8 | CD300e  +  CD303 | CD45 | CD14 | 5 |
|  | cyIL-12 | CD141  BV510 | CD62L | CD33 | HLA-DR | CD16 | FcεRI  FITC | cyIL-13 | cyIL-6 | - | cyIL-10 | CD300e  +  CD303 | CD45 | CD14 |  |

**Abbreviations:** AF700, Alexa Fluor 700; APC, Allophycocyanin; BB, Brilliant Blue; BV, Brilliant Violet, cy, cytoplasmic; CF, Cyanin-based Fluorescent dye; Cy7, Cyanin7; FITC, Fluorescein isothiocyanate; H7, Hilite7; PE, Phycoerythrin; PerCP Cy5.5, Peridinin-chlorophyll-protein-cyanin 5.5; cy, cytoplasmic; OC, Orange Cytognos;

**SUPPLEMENTARY TABLE 3.** Antibody combinations employed for characterization of CD14^+^ HLA-DR^-/dim^ *vs.* classical monocytes in cord blood (n=4).

| BV421 | PacO | BV605 | BV711 | FITC | PerCP Cy5.5 | PE | PE CF594 | PE Cy7 | APC | APC H7 |
| --- | --- | --- | --- | --- | --- | --- | --- | --- | --- | --- |
| CD192 | CD45 | CD62L | HLA-DR | CD1c | CD33 | CD123 | CD34 | CD16 | CD300e | CD14 |
| CD192 | CD45 | CD62L | HLA-DR | CD36 | CD64 | Slan | CD197 | CD16 | CD300e | CD14 |
| CD192 | CD45 | CD62L | HLA-DR | CD36 | CD64 | CD35 | - | CD16 | CD300e | CD14 |
| CD192 | CD45 | CD62L | HLA-DR | CD36 | CD64 | CD11b | - | CD16 | CD300e | CD14 |
| CD192 | CD45 | CD62L | HLA-DR | CD36 | CD64 | CD11c | - | CD16 | CD300e | CD14 |
| CD192 | CD45 | CD62L | HLA-DR | CD36 | CD64 | CD13 | - | CD16 | CD300e | CD14 |
| CD192 | CD45 | CD62L | HLA-DR | CD36 | CD64 | CD33 | - | CD16 | CD300e | CD14 |
| CD192 | CD45 | CD62L | HLA-DR | CD36 | CD64 | CD45RA | - | CD16 | CD300e | CD14 |
| CD192 | CD45 | CD62L | HLA-DR | CD36 | CD64 | CD63 | - | CD16 | CD300e | CD14 |
| CD192 | CD45 | CD62L | HLA-DR | CD36 | CD64 | CD86 | - | CD16 | CD300e | CD14 |
| CD192 | CD45 | CD62L | HLA-DR | CD36 | CD64 | CD163 | - | CD16 | CD300e | CD14 |
| CD192 | CD45 | CD62L | HLA-DR | CD36 | CD64 | CD312 | - | CD16 | CD300e | CD14 |
| CD192 | CD45 | CD62L | HLA-DR | CD36 | CD64 | CD123 | - | CD16 | CD300e | CD14 |
| CD192 | CD45 | CD62L | HLA-DR | cyCD68 | CD64 | Slan | - | CD16 | CD300e | CD14 |
| CD192 | CD45 | CD62L | HLA-DR | CD36 | CD64 | cyMPO | - | CD16 | CD300e | CD14 |

**Abbreviations:** APC, allophycocyanin; BV, Brilliant Violet, cy, cytoplasmic; CF, Cyanin-based Fluorescent dye; Cy7, Cyanin7; FITC, Fluorescein isothiocyanate; H7, Hilite7; PE, Phycoerythrin; PacO, Pacific orange; PerCP Cy5.5, Peridinin-chlorophyll-protein-cyanin 5.5

**SUPPLEMENTARY TABLE 4.** Immunophenotypic profile of the 23 innate myeloid cell (IMC) populations identified employing the 14-color version of the IMC flow cytometry tube (version 4).

| **Populations** | **Immunophenotype** |
| --- | --- |
| **Eosinophils** | CD1c^-*^, CD5^-*^, CD14^-^, CD16^-^, CD33^lo^, CD34^-^, CD36^-**^, CD45^+^, CD62L^+^, CD141^-*^, CD192^-^, CD300e^-^, CD303^-^, FcεRI^-*^, HLA-DR^-^, Slan^-*^ |
| **Neutrophils** | CD1c^-^, CD5^-^, CD14^-^, CD16^-/++^, CD33^+^, CD34^-^, CD36^-**^, CD45^lo/+^, CD62L^-/++^, CD141^-/lo^, CD192^-/+^, CD300e^-^, CD303^-^, FcεRI^-^, HLA-DR^-^, Slan^-^ |
| **Mature neutrophils** | CD1c^-^, CD5^-^, CD14^-^, CD16^++^, CD33^lo^, CD34^-^, CD36^-**^, CD45^+^, CD62L^++^, CD141^-/lo^, CD192^-/+^, CD300e^-^, CD303^-^, FcεRI^-^, HLA-DR^-^, Slan^-^ |
| **Immature neutrophils** | CD1c^-^, CD5^-^, CD14^-^, CD16^-/lo^, CD33^+^, CD34^-^, CD36^-**^, CD45^lo^, CD62L^-/+^, CD141^-^, CD192^-^, CD300e^-^, CD303^-^, FcεRI^-^, HLA-DR^-^, Slan^-^ |
| **CD62L^+^ immature neutrophils** | CD1c^-^, CD5^-^, CD14^-^, CD16^lo^, CD33^+^, CD34^-^, CD36^-**^, CD45^lo^, CD62L^+^, CD141^-^, CD192^-^, CD300e^-^, CD303^-^, FcεRI^-^, HLA-DR^-^, Slan^-^ |
| **CD62L^-^ immature neutrophils** | CD1c^-^, CD5^-^, CD14^-^, CD16^-^, CD33^+^, CD34^-^, CD36^-**^, CD45^lo^, CD62L^-^, CD141^-^, CD192^-^, CD300e^-^, CD303^-^, FcεRI^-^, HLA-DR^-^, Slan^-^ |
| **Basophils** | CD1c^-^, CD5^-^, CD14^-^, CD16^-/+^, CD33^+^, CD34^-^, CD36^-**^, CD45^lo^, CD62L^++^, CD141^-^, CD192^+^, CD300e^-^, CD303^-^, FcεRI^++^, HLA-DR^-^, Slan^-^ |
| **Monocytes** | CD1c^lo^, CD5^-^, CD14^-/+^, CD16^-^, CD33^+/++^, CD34^-^, CD36^-/+^, CD45^+^, CD62L^-/+^, CD141^lo^, CD192^-/+^, CD300e^lo/++^, CD303^-^, FcεRI^-/+^, HLA-DR^+/++^, Slan^-/+^ |
| **cMo** | CD1c^lo^, CD5^-^, CD14^+^, CD16^-^, CD33^++^, CD34^-^, CD36^+^, CD45^+^, CD62L^-/+^, CD141^lo^, CD192^+^, CD300e^lo^, CD303^-^, FcεRI^-^, HLA-DR^+/++^, Slan^-^ |
| **CD62L^+^ FcεRI^-^ cMo** | CD1c^lo^, CD5^-^, CD14^+^, CD16^-^, CD33^++^, CD34^-^, CD36^+^, CD45^+^, CD62L^+^, CD141^lo^, CD192^+^, CD300e^lo^, CD303^-^, FcεRI^-^, HLA-DR^+^, Slan^-^ |
| **CD62L^+^ FcεRI^+^ cMo** | CD1c^lo^, CD5^-^, CD14^+^, CD16^-^, CD33^++^, CD34^-^, CD36^+^, CD45^+^, CD62L^+^, CD141^lo^, CD192^+^, CD300e^lo^, CD303^-^, FcεRI^+^, HLA-DR^+^, Slan^-^ |
| **CD62L^-^ FcεRI^-^ cMo** | CD1c^lo^, CD5^-^, CD14^+^, CD16^-^, CD33^++^, CD34^-^, CD36^+^, CD45^+^, CD62L^-^, CD141^lo^, CD192^+^, CD300e^lo^, CD303^-^, FcεRI^-^, HLA-DR^++^, Slan^-^ |
| **CD62L^-^ FcεRI^+^ cMo** | CD1c^lo^, CD5^-^, CD14^+^, CD16^-^, CD33^++^, CD34^-^, CD36^+^, CD45^+^, CD62L^-^, CD141^lo^, CD192^+^, CD300e^lo^, CD303^-^, FcεRI^+^, HLA-DR^++^, Slan^-^ |
| **iMo** | CD1c^lo^, CD5^-^, CD14^+^, CD16^+^, CD33^++^, CD34^-^, CD36^+^, CD45^+^, CD62L^-/lo^, CD141^+^, CD192^+^, CD300e^+^, CD303^-^, FcεRI^-^, HLA-DR^++^, Slan^-^ |
| **ncMo** | CD1c^lo^, CD5^-^, CD14^-/lo^, CD16^+^, CD33^+/++^, CD34^-^, CD36^-/+^, CD45^++^, CD62L^-^, CD141^+^, CD192^-/+^, CD300e^++^, CD303^-^, FcεRI^-^, HLA-DR^+^, Slan^-/+^ |
| **CD36^+^ Slan^-^ ncMo** | CD1c^lo^, CD5^-^, CD14^lo^, CD16^+^, CD33^++^, CD34^-^, CD36^+^, CD45^++^, CD62L^-^, CD141^+^, CD192^-/+^, CD300e^++^, CD303^-^, FcεRI^-^, HLA-DR^+^, Slan^-^ |
| **CD36^-^ Slan^-^ ncMo** | CD1c^lo^, CD5^-^, CD14^-^, CD16^+^, CD33^++^, CD34^-^, CD36^-^, CD45^++^, CD62L^-^, CD141^+^, CD192^-^, CD300e^++^, CD303^-^, FcεRI^-^, HLA-DR^+^, Slan^-^ |
| **CD36^+^ Slan^+^ ncMo** | CD1c^lo^, CD5^-^, CD14^-^, CD16^+^, CD33^+^, CD34^-^, CD36^+^, CD45^++^, CD62L^-^, CD141^+^, CD192^-^, CD300e^++^, CD303^-^, FcεRI^-^, HLA-DR^+^, Slan^+^ |
| **CD36^-^ Slan^+^ ncMo** | CD1c^lo^, CD5^-^, CD14^-^, CD16^+^, CD33^+^, CD34^-^, CD36^-^, CD45^++^, CD62L^-^, CD141^+^, CD192^-^, CD300e^++^, CD303^-^, FcεRI^-^, HLA-DR^+^, Slan^+^ |
| **M-MDSC** | CD1c^lo^, CD5^-^, CD14^lo/+^, CD16^-/+^, CD33^+^, CD34^-^, CD36^-/+^, CD45^+^, CD62L^-/+^, CD141^-^, CD192^-/lo^, CD300e^-/lo^, CD303^-^, FcεRI^-^, HLA-DR^-/lo^, Slan^-^ |
| **CD1c^+^ myDCs** | CD1c^lo/+^, CD5^-/+^, CD14^-/lo^, CD16^-^, CD33^++^, CD34^-^, CD36^+^, CD45^+^, CD62L^-/+^, CD141^lo/+^, CD192^+^, CD300e^-/+^, CD303^-^, FcεRI^+^, HLA-DR^++^, Slan^-^ |
| **CD1c^+^ CD14^lo^ myDCs** | CD1c^lo^, CD5^-^, CD14^lo^, CD16^-^, CD33^++^, CD34^-^, CD36^+^, CD45^+^, CD62L^-/+^, CD141^lo^, CD192^+^, CD300e^-/+^, CD303^-^, FcεRI^+^, HLA-DR^++^, Slan^-^ |
| **CD1c^+^ CD14^-^ CD5^-^ myDCs** | CD1c^+^, CD5^-^, CD14^-^, CD16^-^, CD33^++^, CD34^-^, CD36^+^, CD45^+^, CD62L^-/+^, CD141^lo^, CD192^+^, CD300e^-/+^, CD303^-^, FcεRI^+^, HLA-DR^++^, Slan^-^ |
| **CD1c^+^ CD14^-^ CD5^+^ myDCs** | CD1c^+^, CD5^+^, CD14^-^, CD16^-^, CD33^++^, CD34^-^, CD36^+^, CD45^+^, CD62L^-/+^, CD141^lo/+^, CD192^+^, CD300e^-/+^, CD303^-^, FcεRI^+^, HLA-DR^++^, Slan^-^ |
| **CD141^+^ myDCs** | CD1c^-^, CD5^-/lo^, CD14^-^, CD16^-^, CD33^++^, CD34^-^, CD36^-*^, CD45^+^, CD62L^+^, CD141^++^, CD192^+^, CD300e^-^, CD303^-^, FcεRI^-^, HLA-DR^++^, Slan^-^ |
| **pDCs** | CD1c^-^, CD5^-^, CD14^-^, CD16^-^, CD33^-^, CD34^-^, CD36^+^, CD45^+^, CD62L^-/+^, CD141^+^, CD192^+^, CD300e^-^, CD303^+^, FcεRI^lo/+^, HLA-DR^++^, Slan^-^ |
| **Axl^+^ DCs** | CD1c^-^, CD5^-/+^, CD14^-^, CD16^-^, CD33^+^, CD34^-^, CD36^lo^, CD45^+^, CD62L^lo/+^, CD141^+^, CD192^+^, CD300e^-^, CD303^+^, FcεRI^-/+^, HLA-DR^++^, Slan^-^ |
| **CD34^+^ HPC** | CD1c^-^, CD5^-^, CD14^-^, CD16^-^, CD33^-/+^, CD34^+^, CD36^-/+^, CD45^+^, CD62L^lo/+^, CD141^-^, CD192^lo^, CD300e^-^, CD303^-^, FcεRI^-/lo^, HLA-DR^+^, Slan^-^ |
| **preDC** | CD1c^-^, CD5^-^, CD14^-^, CD16^-^, CD33^-/+^, CD34^lo^, CD36^-**^, CD45^+^, CD62L^+^, CD141^-^, CD192^lo^, CD300e^-^, CD303^-^, FcεRI^-^, HLA-DR^++^, Slan^-^ |

*, cell population displays autofluorescence in the channel employed for the study of the maker; **, expression of CD36 can be observed in (part) of the population due to adhesion of platelets to the cells.

**Abbreviations:** cMo, classical monocytes; iMo, intermediate monocytes; ncMo, non-classical monocytes; DCs, dendritic cells; myDC, myeloid DCs; pDC, plasmacytoid DCs.

**SUPPLEMENTARY TABLE 5.** Intra**-**assay reproducibility of technical replicates for the identification of cell populations in K3 ethylenediaminetetraacetic acid (EDTA)-anticoagulated peripheral blood samples (n=5) stained with version 3 of the EuroFlow Innate Myeloid Cell (IMC) tube.

| **Populations** | **Absolute count**  **(cells/μL)**  [median (min-max)] | **% intra-CV**  [median (min-max)] |
| --- | --- | --- |
| Unidentified cells | 2134.2 (1474.3 – 2555.6) | 2.3 (0.2 – 4.6) |
| Eosinophils | 121.2 (55.4 – 277.5) | 1.9 (0.06 – 7.8) |
| Neutrophils | 3013.6 (2819.6 – 3345.0) | 1.5 (0.05 – 4.2) |
| Mature neutrophils | 3009.6 (2815.8 – 3342.3) | 1.5 (0.05 – 4.1) |
| Immature neutrophils | 3.8 (1.5 – 3.9) | 4.7 (0.5 – 11.5) |
| CD62L+ immature neutrophils | 0.4 (0.2 – 8.8) | 10.3 (5.1 – 17.9) |
| CD62L- immature neutrophils | 3.1 (1.1 – 3.5) | 3.8 (0.2 – 9.2) |
| Basophils | 38.1 (33.3 – 46.0) | 1.2 (0.4 – 3.0) |
| Monocytes | 357.5 (288.5 – 458.9) | 0.5 (0.3 -2.9) |
| cMo | 315.6 (249.8 – 385.4) | 1.1 (0.04 – 2.7) |
| CD62L+ FcεRI- cMo | 158.4 (117.5 – 342.6) | 1.9 (0.5 -12.5) |
| CD62L+ FcεRI+ cMo | 109.4 (6.0 – 172.5) | 6.9 (0.6 – 20.3) |
| CD62L- FcεRI- cMo | 23.7 (11.8 – 76.5) | 12.7 (5.3 – 16.3) |
| CD62L- FcεRI+ cMo | 8.9 (0.8 – 36.7) | 5.4 (0.6 – 32.5) |
| iMo | 15.6 (7.3 – 22.5) | 11.1 (3.0 – 16.7) |
| ncMo | 36.4 (26.9 – 51.0) | 1.2 (0.5 – 4.6) |
| CD36+ Slan- ncMo | 10.8 (7.8 – 14.5) | 9.4 (0.5 – 17.3) |
| CD36- Slan- ncMo | 1.8 (0.5 – 8.0) | 16.3 (0.0 – 19.5) |
| CD36+ Slan+ ncMo | 1.0 (0.5 – 1.8) | 3.0 (0.0 – 18.7) |
| CD36- Slan+ ncMo | 22.9 (14.3 – 28.6) | 3.0 (1.7 – 5.6) |
| CD1c+ myDCs | 16.5 (11.2 – 19.9) | 2.7 (0.8 – 8.8) |
| CD1c+ CD14- myDCs | 11.7 (8.3 – 15.2) | 2.8 (0.8 – 6.1) |
| CD1c+/CD14lo myDCs | 4.7 (2.9 – 4.8) | 7.7 (0.0 – 15.8) |
| CD141+ myDCs | 1.0 (6.6 – 15.7) | 1.5 (0.0 – 6.5) |
| pDCs | 9.7 (6.6 – 15.7) | 2.9 (0.9 – 6.4) |
| Axl^+^ DCs | 0.7 (0.3 – 0.8) | 13.0 (8.4 – 33.3) |
| **Average intra-assay %CV for all populations**  **identified in the tube (mean±standard deviation)** | | **5.0% ± 4.5%** |

Populations with %intra-assay CV >10% and 15% are highlighted with a lighter and darker shade of of gray, respectively.

**Abbreviations:** cMo, classical monocytes; iMo, intermediate monocytes; ncMo, non-classical monocytes; DCs, dendritic cells; myDC, myeloid DCs; pDC, plasmacytoid DCs.

**SUPPLEMENTARY TABLE 6.** Intra**-**assay reproducibility of manual analysis performed twice by the same analyst at different timepoints (≥2 months apart) for the identification of cell populations in K3 ethylenediaminetetraacetic acid (EDTA)-anticoagulated peripheral blood samples (n=6) stained with version 3 of the EuroFlow Innate Myeloid Cell (IMC) tube.

| **Populations** | **Relative frequency from nucleated cells**  **(%)**  [median (min-max)] | **% intra-assay CV**  [median (min-max)] |
| --- | --- | --- |
| Unidentified cells | 33.9 (31.2 - 43.1) | 0.09 (0.01-1.1) |
| Eosinophils | 3.0 (1.8 - 11.3) | 0.3 (0.1-1.0) |
| Neutrophils | 51.8 (35.5 – 59.0) | 0.08 (0.02-1.1) |
| Mature neutrophils | 51.8 (35.5 – 59.0) | 0.07 (0.02-1.1) |
| Immature neutrophils | 0.06 (0.02 – 0.21) | 0.03 (0.0-0.5) |
| CD62L+ immature neutrophils | 0.01 (0.004 – 0.04) | 0.08 (0.0-0.5) |
| CD62L- immature neutrophils | 0.04 (0.01 – 0.17) | 0.02 (0.0-0.4) |
| Basophils | 0.7 (0.4 – 1.5) | 0.9 (0.04-4.4) |
| Monocytes | 9.2 (6.2 - 12.4) | 0.5 (0.05-1.7) |
| cMo | 8.0 (5.5 – 9.9) | 0.6 (0.02-2.8) |
| CD62L+ FcεRI- cMo | 5.4 (3.7 – 8.5) | 9.0 (3.1-16.8) |
| CD62L+ FcεRI+ cMo | 0.8 (0.08 – 1.9) | 3.0 (0.2-53.0) |
| CD62L- FcεRI- cMo | 0.9 (0.4 – 1.0) | 36.7 (21.7-171.3) |
| CD62L- FcεRI+ cMo | 0.2 (0.02 – 0.5) | 37.7 (5.8-95.0) |
| iMo | 0.4 (0.2 – 1.0) | 8.0 (0.4-20.2) |
| ncMo | 0.8 (0.5 – 2.1) | 4.5 (1.1-13.3) |
| CD36+ Slan- ncMo | 0.2 (0.10 – 0.4) | 12.6 (0.02-28.0) |
| CD36- Slan- ncMo | 0.2 (0.07 – 0.4) | 40.0 (9.0-68.6) |
| CD36+ Slan+ ncMo | 0.01 (0.006 – 0.14) | 2.6 (0.4-17.2) |
| CD36- Slan+ ncMo | 0.2 (0.1 – 1.3) | 11.1 (0.3-17.2) |
| CD1c+ myDCs | 0.3 (0.2 – 0.5) | 4.9 (0.4-13.7) |
| CD1c+ CD14- myDCs | 0.2 (0.1 – 0.4) | 1.4 (0.06-3.0) |
| CD1c+ CD14lo myDCs | 0.09 (0.05 – 0.2) | 9.2 (0.2-30.1) |
| CD141+ myDCs | 0.01 (0.004 - 0.03) | 3.3 (0.2-5.2) |
| pDCs | 0.2 (0.03 – 0.5) | 2.1 (0.5-12.4) |
| Axl^+^ DCs | 0.01 (0.004 – 0.03) | 33.9 (0.9-108.2) |
| **Average intra-assay %CV for all populations**  **identified in the tube (mean±standard deviation)** | | 8.0% ± 12.7% |

Populations with %intra-assay CV >10% and 15% are highlighted with a lighter and darker shade of of gray, respectively.

**Abbreviations:** CV, coefficient of variation; cMo, classical monocytes; iMo, intermediate monocytes; ncMo, non-classical monocytes; DCs, dendritic cells; myDCs, myeloid DCs; pDCs, plasmacytoid DCs.

**
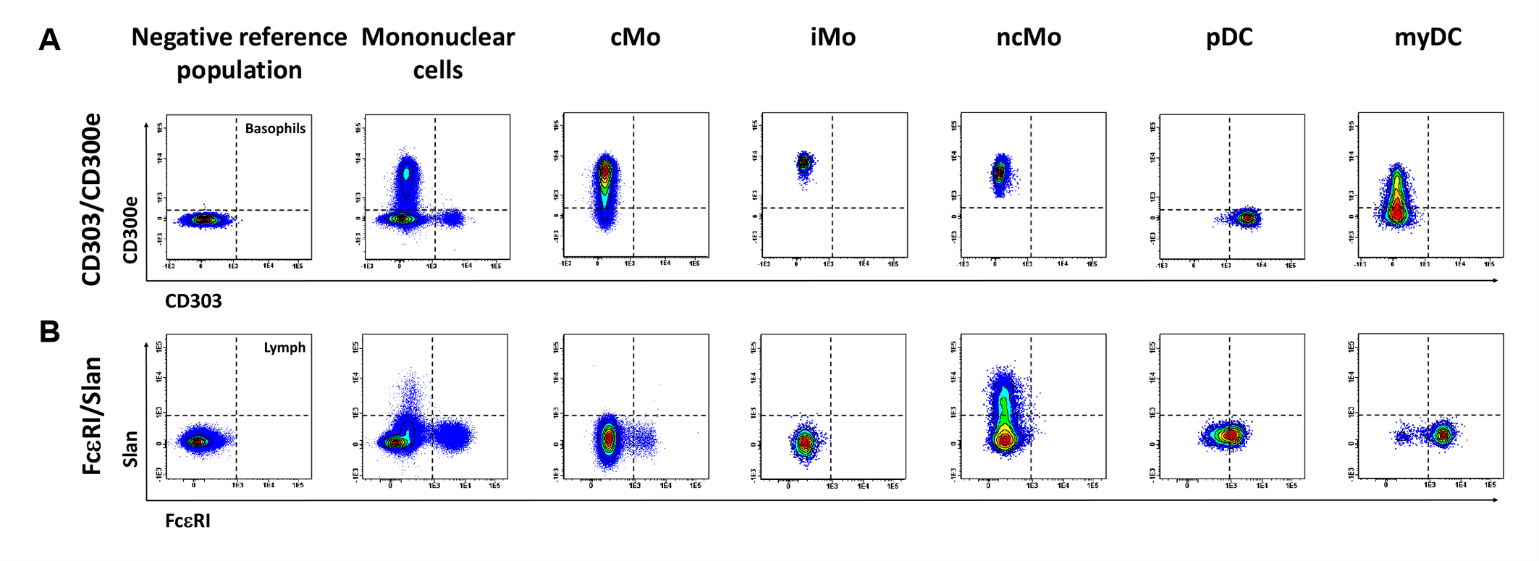
**

**Supplementary Figure 1: Expression pattern of CD300e, CD303, FcεRI and Slan.** Dot plots of a representative sample depicting the excluding expression patterns of CD300e and CD303 (Panel A) and FcεRI and Slan (Panel B) on mononuclear cell populations (monocytic and dendritic cell populations). cMo, classical monocytes; iMo, intermediate monocytes; ncMo, non-classical monocytes; pDC, plasmacytoid dendritic cells; myDC, myeloid dendritic cells; Lymph, lymphocytes.


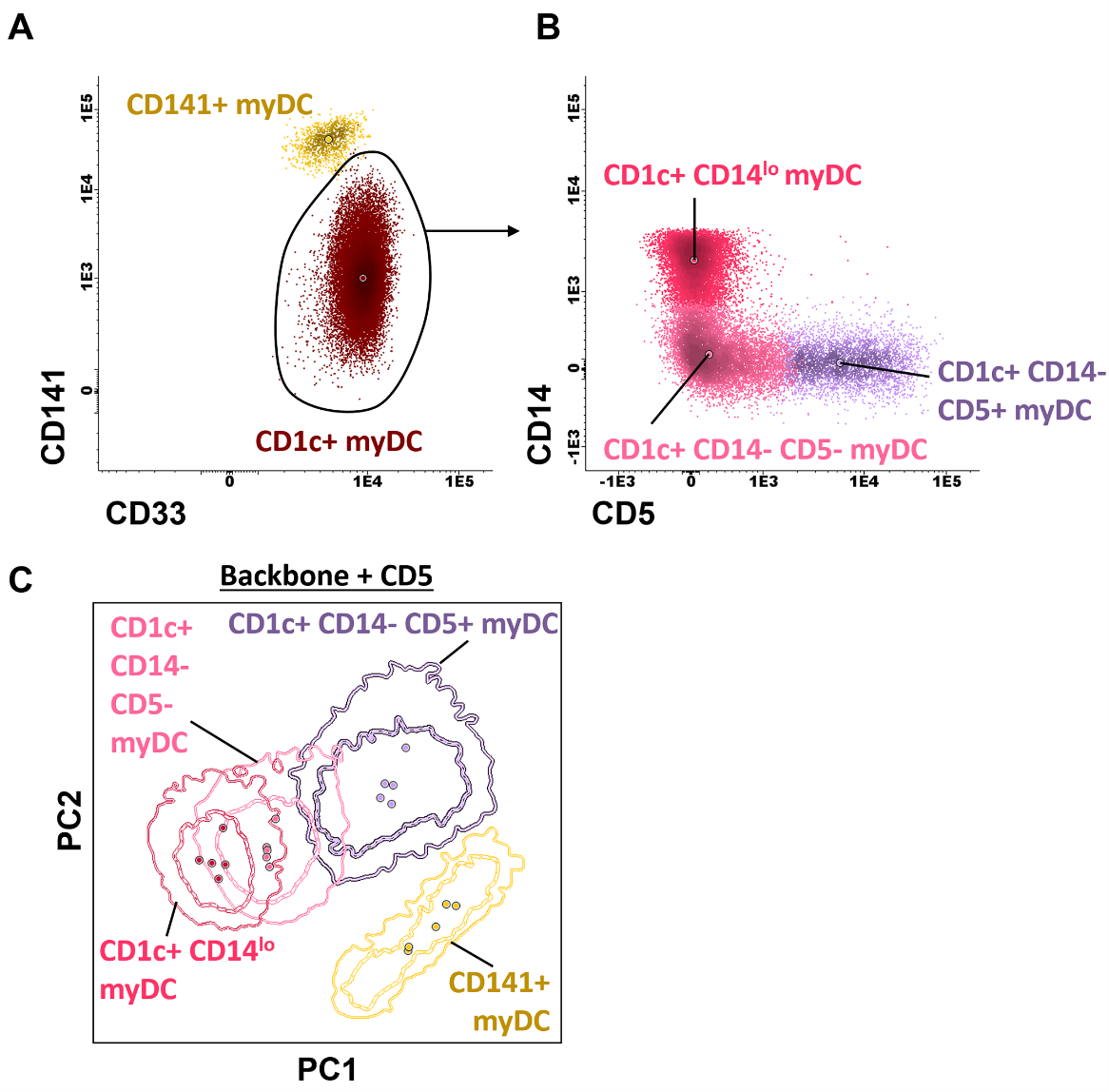


**Supplementary Figure 2: Subsetting of CD1c+ myeloid dendritic cells (myDCs).** Identification of the major myDC populations is depicted in panel A. Pro-inflammatory CD1c+ CD14^lo^ myDCs can be identified with the backbone combination and inclusion of CD5 allows for further subsetting of myDC CD1c^+^ CD14^-^ in CD5^+^ and CD5^-^ populations (panel B). Principal component analysis (PCA) plot shown in panel C represents separation of all myDC populations in 5 donors when backbone markers (CD1c, CD14, CD16, CD33, CD45, CD141, CD300e + CD303, HLA-DR) and CD5 are included. Solid circles represent median values for the parameters evaluated, inner dotted and outer solid lines depict the first standard and second standard deviations for each population identified, respectively. myDC, myeloid dendritic cells; PC, principal component.

**
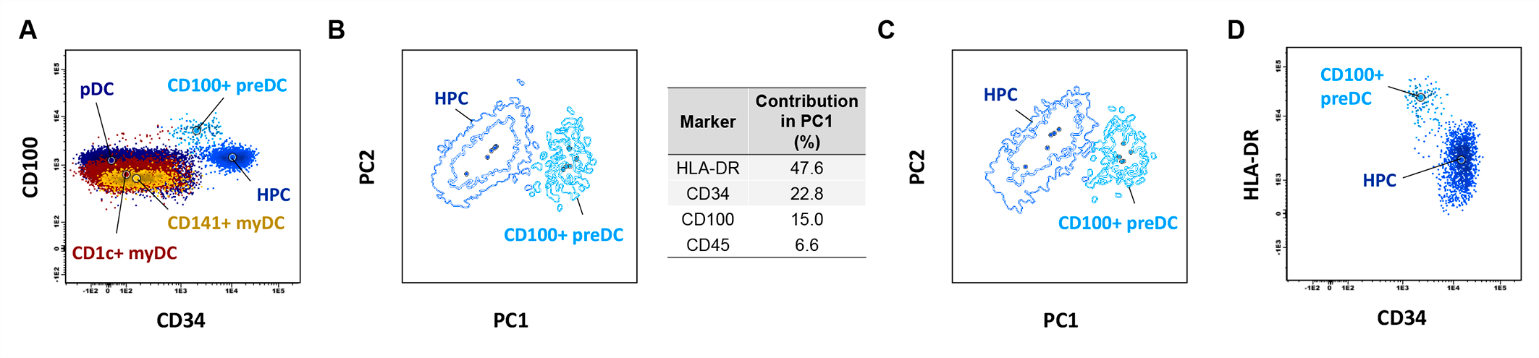
**

**Supplementary Figure 3. Strategy for identification of CD100^hi^ CD34^int^ dendritic cell precursors (preDCs).** Representative dot plot depicting the identificiation of preDCs based on expression of CD34 and CD100 is shown in Panel A. Panel B conveys the marker performance for identification of hematopoietic precursor cells (HPCs) *vs.* preDCs as determined employing principal component analysis (PCA) in the context of cross-staining with CD14, CD16, CD33, CD34, CD45, CD100, CD300e, CD303 and HLA-DR (n=5). The relative weight of the highest contributing markers (>5%) for population discrimination is depicted in the table. Panel C shows the separation performance of HPCs *vs.* preDCs using only the markers showing the highest contribution in panel B (CD34 and HLA-DR). Identification of preDC based on expression of CD34 and HLA-DR is shown in a representative dot plot (Panel D). Solid circles represent median values for the parameters evaluated, inner dotted and outer solid lines depict the first standard and second standard deviations for each population identified. myDC, myeloid dendritic cells; pDC, plasmacytoid dendritic cells; HPC, hematopoietic precursor cells; PC, principal component.


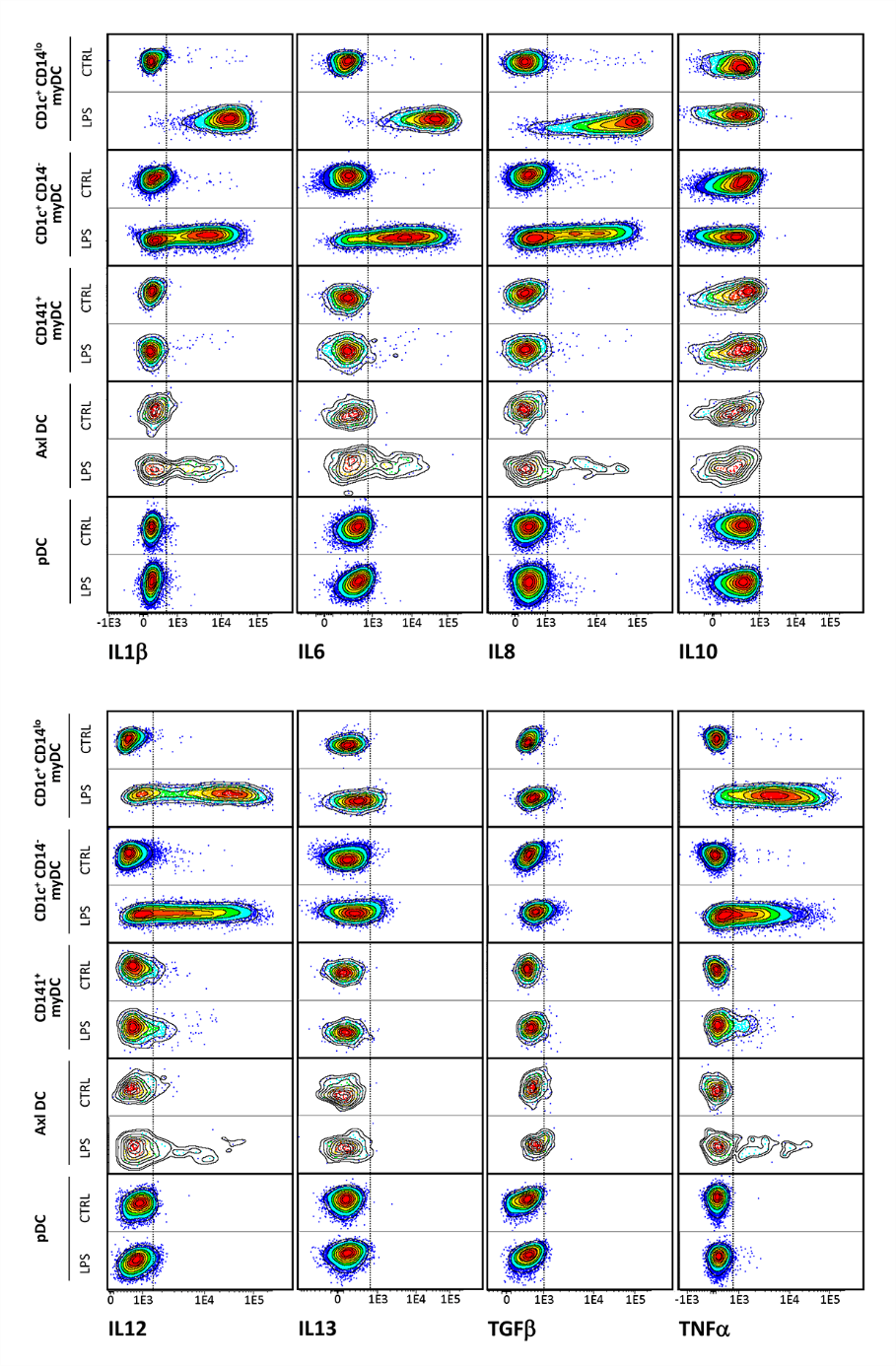


**Supplementary Figure 4. Representative plots of the intracellular cytokine staining expression patterns of the different dendritic cell (DC) populations in steady-state and upon stimulation with 100 ng/ml of lipopolysaccharide (LPS).**

CTRL, unstimulated control; LPS, lipopolysaccharide; DC, dendritic cells; myDC, myeloid dendritic cells; pDC, plasmacytoid dendritic cells. IL, interleukin; TGF, transforming growth factor; TNF, tumor necrosis factor.

**
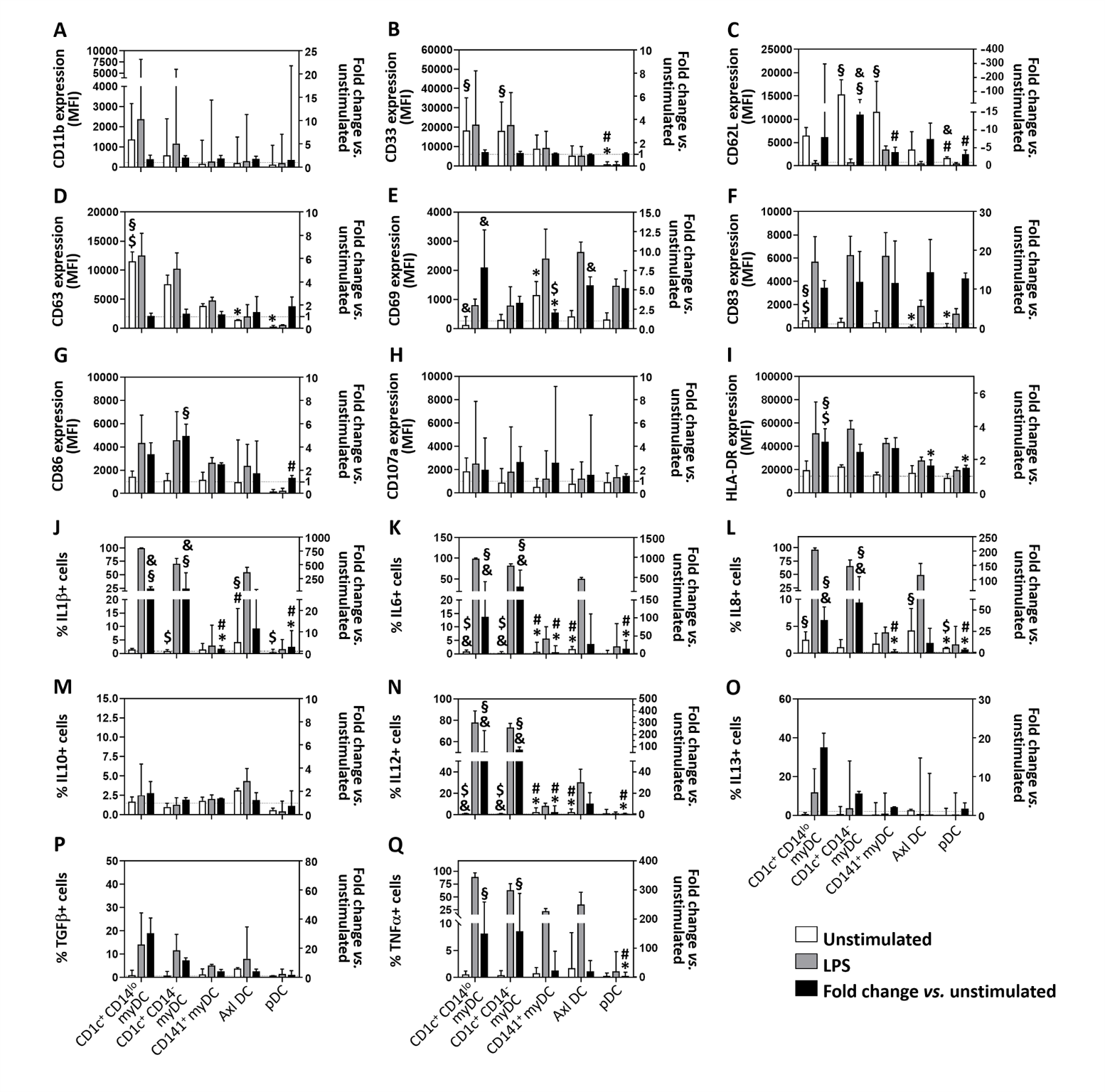
**

**Supplementary Figure 5. Expression of membrane activation/maturation markers and cytokine secretion patterns of the different dendritic cell (DC) populations in steady-state and upon stimulation with 100 ng/ml of lipopolysaccharide (LPS).** Surface expression pattern of activation and maturation-related markers (panels A-I) and cytokine production profile (panels J-Q) of the distinct DC subsets, identified employing the combined antibody backbone selected for their immunophenotypic identification (CD1c, CD14, CD16, CD33, CD45, CD141, CD300e, CD303 and HLA-DR). Bars indicate median plus 95% confidence interval, white bars reflect the steady-state condition, whereas the grey bars depict the protein expression upon stimulation with 100ng/mL of LPS for 6h, and the black bars represent the fold change of the stimulated condition *vs.* unstimulated. Horizontal dotted line indicates the fold change of 1. myDC, myeloid dendritic cells; pDC, plasmacytoid dendritic cells; MFI, mean fluorescence intensity; LPS, lipopolysaccharide. Statistical differences were evaluated employing Kruskall-Wallis and Wilcoxon tests to compare between populations and steady-state *vs.* stimulation, respectively. P value <0.05 and false discovery rate (FDR) of 5% *vs.* * CD1c^+^ CD14^lo^ myDC , ^#^ CD1c^+^ CD14^-^ myDC, ^&^ CD141^+^ myDC, ^$^Axl^+^ DC, ^§^pDC.


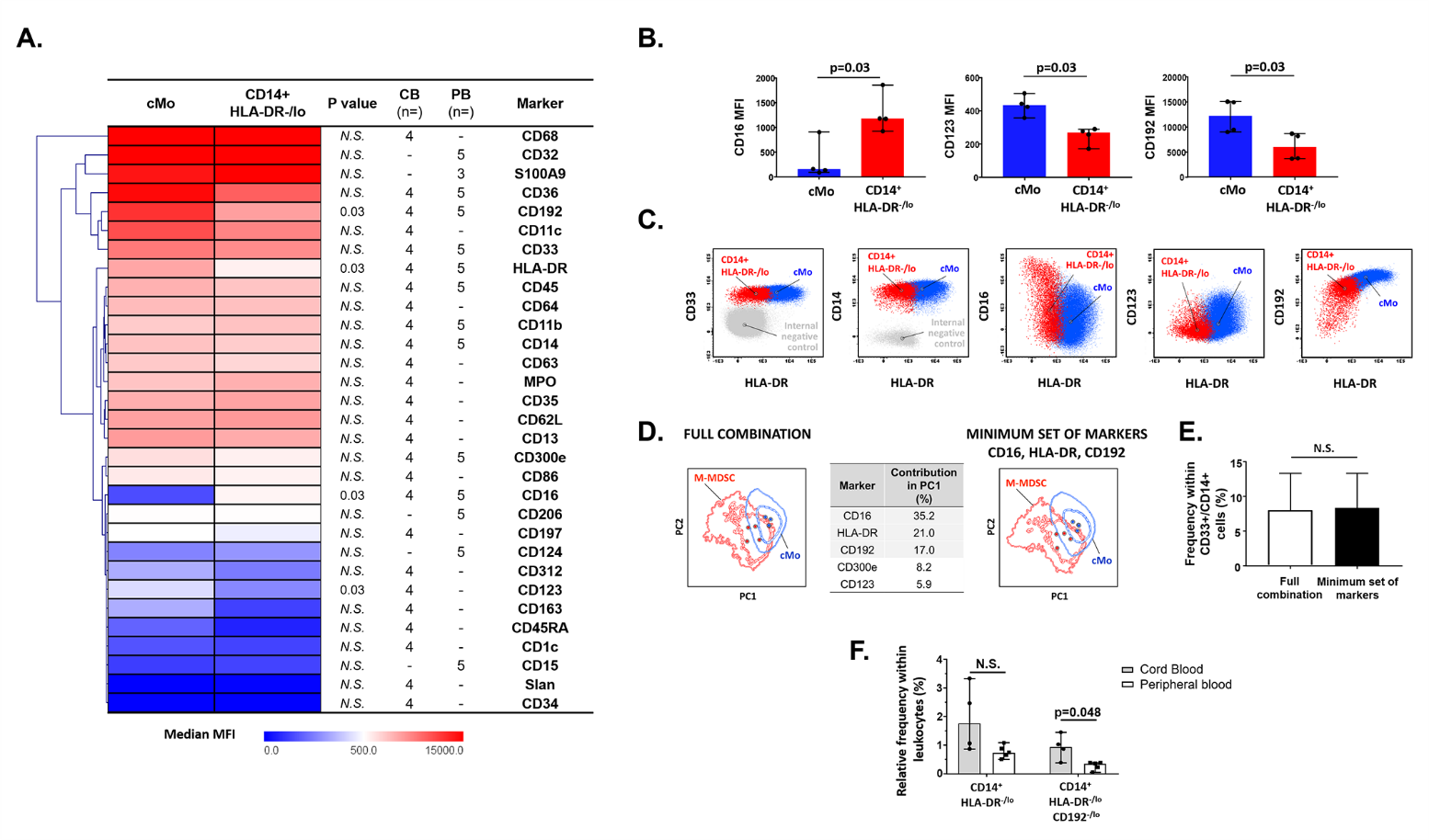


**Supplementary Figure 6. Selection of the set of markers for identification of monocytic myeloid-derived suppressor cells (M-MDSCs)**. Hierarchical clustering analysis of the expression of monocytic and M-MDSC-related markers in classical monocytes (cMos) *vs.* CD14^+^ HLA-DR^-/lo^ cells (Panel A). Expression pattern of proteins with significantly different expression between the two populations is depicted in Panels B and C. Selection of the minimum set of markers for reliable identification of M-MDSCs *vs.* cMos employing principal component analysis (PCA) including the complete set of markers differently expressed (CD14, CD16, CD33, CD45, CD123, CD192, CD300e and HLA-DR in combination with the size and internal complexity information) or the minimum set of markers (CD16, CD192 and HLA-DR) is depicted in Panel D. Panel E shows the performance of the full combination *vs.* the minimum set of markers in the relative frequency of M-MDSCs within CD33^+^/CD14^+^ cells (n=4), whereas Panel F depicts the relative frequency within leukocytes of M-MDSCs defined based only on CD14^+^ HLA-DR^-/lo^ *vs.* CD14^+^ HLA-DR^-/lo^ CD192^-/lo^ in cord blood and adult peripheral blood. Statistical differences were evaluated employing Wilcoxon and Mann-Whitney tests, to compare between gating strategies and CB vs. PB, respectively. Solid circles in the PCA plot represent median values for the parameters evaluated, inner dotted and outer solid lines depict the first standard and second standard deviations for each population identified. Bars on graphs depict the median and 95% confidence interval. cMo, classical monocytes; CB, cord blood; PB, peripheral blood; MFI, mean fluorescence intensity; PC, principal component. N.S., not statistically significant (p value>0.05).


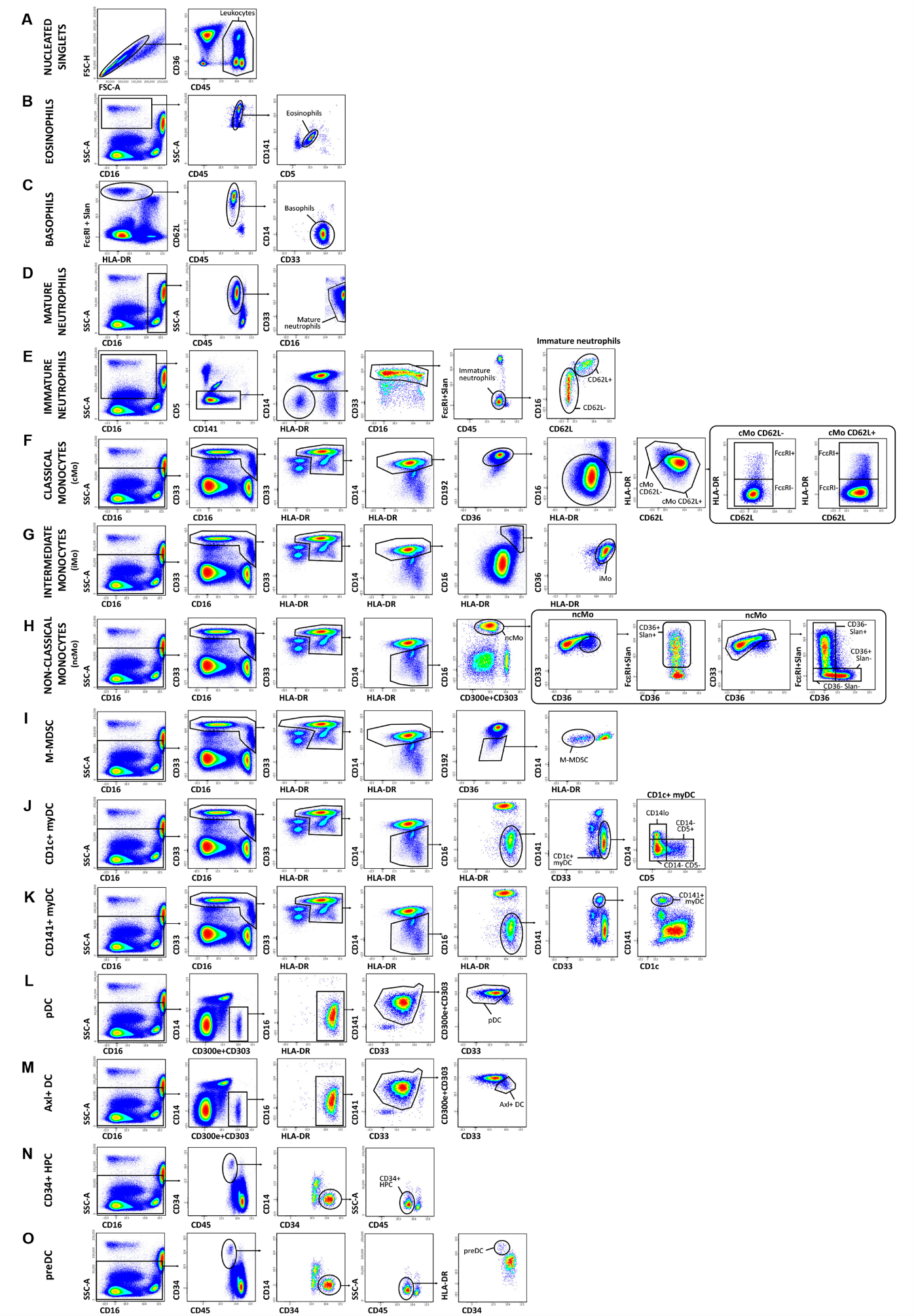


Supplementary Figure 7: Gating strategy for identification of the 23 innate myeloid cell (IMC) populations employing the 14-color version of the IMC flow cytometry tube (version 4). Removal of doublets and debris is shown in panel A. Panels B-O depict the strategy employed for identification of each IMC population, gated on leukocytes identified in panel A.

cMo, classical monocytes; iMo, intermediate monocytes; ncMo, non-classical monocytes; DC, dendritic cell; myDC, myeloid dendritic cells; pDC, plasmacytoid dendritic cells; HPC, hematopoietic precursor cells; M-MDSC; monocytic myeloid-derived suppressor cells.


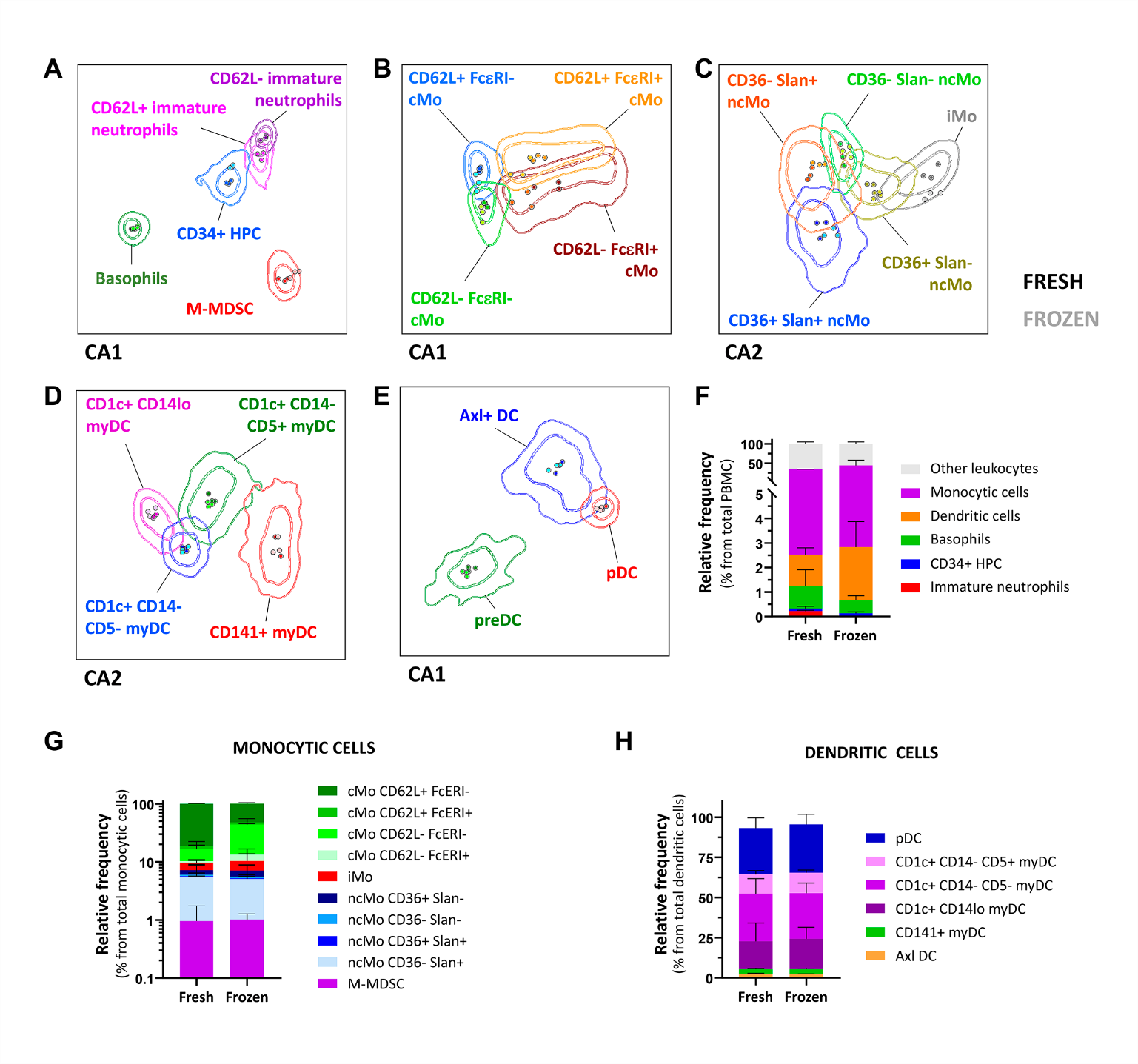


**Supplementary Figure 8: Performance of the EuroFlow innate myeloid cell (IMC) 14-color extended combination (version 4) for the staining of fresh *vs.* frozen peripheral blood mononuclear cells (PBMCs) (n=3).**

Canonical multivariate analysis (CA) plots depicting the impact of freezing (shown as different shades of the population color) on the immunophenotypic patterns *vs.* PBMCs stained immediately upon collection and isolation are depicted in Panels A-E. Impact of freezing on the relative frequency within total PBMCs of the major leukocyte populations is depicted in panel F. Panels G and H exhibit the relative frequency of monocytic and dendritic cell populations within total monocytes and total dendritic cells, respectively. Solid circles in all CA plots represent median values for the parameters evaluated in each sample, inner dotted and outer solid lines depict the first standard and second standard deviations for each population identified in the fresh condition. Data in the frequency plots is reported as median and 95% confidence interval. cMo, classical monocytes; iMo, intermediate monocytes; ncMo, non-classical monocytes; DC, dendritic cells; pDC, plasmacytoid dendritic cells; myDC, myeloid dendritic cells; HPC, hematopoietic precursor cells; M-MDSC, monocytic-myeloid derived suppressor cells; CA, canonical multivariate analysis.


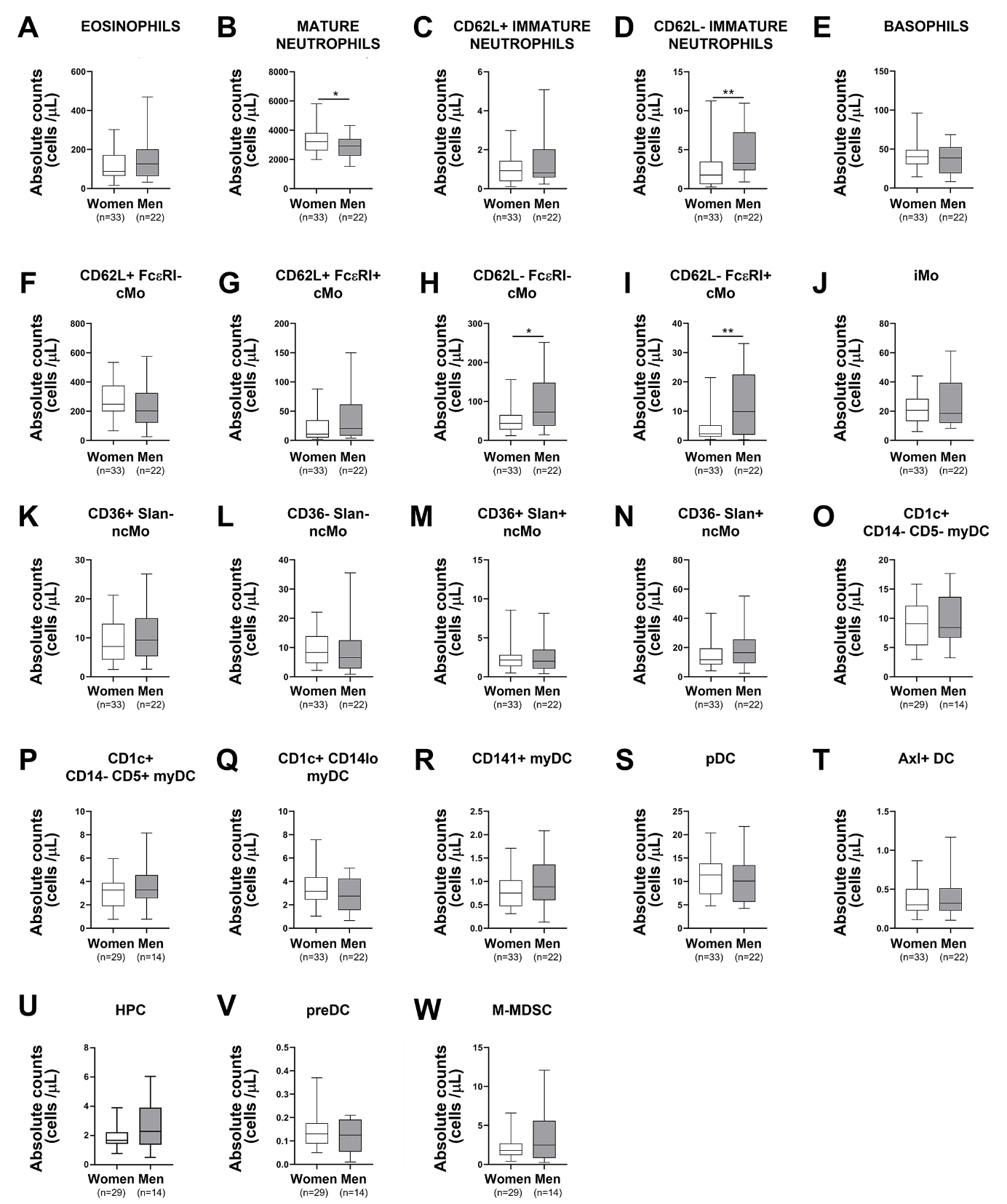


**Supplementary Figure 9:** **Gender-related difference in the distribution of innate myeloid cell populations in peripheral blood of healthy young adults (ages 18-39 years).** Granulocytes are depicted in panels A-E. Panels F-N show monocytic subsets, while dendritic cells (DC) are shown in panels O-T. Hematopoietic precursor cells (HPC), preDC and monocytic myeloid-derived supressor cells (M-MDSC) are represented in panels U, V and W, respectively. *p value <0.05 evaluated employing the Mann-Whitney statistical test. cMo, classical monocytes; iMo, intermediate monocytes; ncMo, non-classical monocytes; DC, dendritic cells; myDC, myeloid dendritic cells; pDC, plasmacytoid dendritic cells; HPC, hematopoietic precursor cells; M-MDSC; monocytic-myeloid derived suppressor cells.
